# An innate IL-25-ILC2-MDSC axis creates a cancer-permissive microenvironment for *Apc*-mutation-driven intestinal tumorigenesis

**DOI:** 10.1126/sciimmunol.abn0175

**Published:** 2022-06-03

**Authors:** Eric Jou, Noe Rodriguez-Rodriguez, Ana-Carolina F. Ferreira, Helen E. Jolin, Paula A. Clark, Kovilen Sawmynaden, Michelle Ko, Jane E. Murphy, Jonathan Mannion, Christopher Ward, David J. Matthews, Simon J. A. Buczacki, Andrew N. J. McKenzie

**Affiliations:** 1 MRC Laboratory of Molecular Biology, Cambridge, CB2 0QH, United Kingdom; 2 LifeArc, SBC Innovation Campus, Stevenage, SG1 2FX. United Kingdom; 3 Cambridge Stem Cell Institute, Jeffrey Cheah Biomedical Centre, Cambridge, CB2 0AW United Kingdom

## Abstract

Interleukin-25 and group 2 innate lymphoid cells (ILC2s) defend the host against intestinal helminth infection, and are associated with inappropriate allergic reactions. IL-33-activated ILC2s were previously found to augment protective tissue-specific pancreatic cancer immunity. Here, we showed that intestinal IL-25-activated ILC2s created an innate cancer-permissive microenvironment. Colorectal cancer (CRC) patients with higher tumor *IL25* expression had reduced survival, and increased IL-25R-expressing tumor-resident ILC2s and myeloid-derived suppressor cells (MDSCs) associated with impaired anti-tumor responses. Ablation of IL-25-signalling reduced tumors, virtually doubling life-expectancy in an *Apc-*mutation-driven model of spontaneous intestinal tumorigenesis. Mechanistically, IL-25 promoted intratumoral ILC2s, which sustained tumor-infiltrating MDSCs to suppress anti-tumor immunity. Therapeutic antibody-mediated blockade of IL-25-signalling decreased intratumoral ILC2s, MDSCs and adenoma/adenocarcinoma, while increasing anti-tumor adaptive T cell and IFNγ-mediated immunity. Thus, the roles of innate epithelium-derived cytokines IL-25 and IL-33, and ILC2s in cancer cannot be generalized. The pro-tumoral nature of the IL-25-ILC2 axis in CRC highlights this pathway as a novel therapeutic target against CRC.

## Introduction

ILC2s are innate immune sentinels within barrier tissues, responding rapidly to acute immune perturbation by releasing effector cytokines including IL-4, IL-5 and IL-13, and promoting a type 2 inflammatory environment (1, 2). At mucosal barriers, ILC2s are activated by alarmin cytokine cues such as IL-25 and IL-33. In the intestine, IL-25 is expressed by tuft cells and is a key factor in ILC2 activation in protective immune responses to parasitic helminth infections (1–4), facilitating the interface between innate and adaptive immunity, and promoting tissue repair following damage. However, the tissue-specific microenvironmental cues that differentially regulate innate ILC2-mediated immunity, especially in the intestine in the context of a chronic cancer-permissive niche, are poorly understood.

Colorectal cancer (CRC) is the most common intestinal cancer in humans, and the second leading cause of cancer-related death worldwide (5). Of the different subtypes of CRC, the most common subgroup (80%) is characterized by causative mutations in the adenomatous polyposis coli *(APC)* tumor suppressor gene, with the loss of *APC* initiating tumor development (6, 7). Additionally, patients who inherit germline mutations in *APC* are susceptible to spontaneous tumor development, mostly in the colon, but also throughout the gastrointestinal tract including in the duodenum and stomach with variable penetrance (8). The development of *Apc*-mutant mouse models has facilitated the study of *Apc*-mutation-mediated spontaneous intestinal tumorigenesis, promoting mechanistic understanding of the immune microenvironment and therapeutic discovery (9–12). These spontaneous models avoid surgery-induced inflammation associated with orthotopic CRC organoid transfer into the colon (13), allowing study of the unperturbed immune response during the onset and development of autochthonous tumorigenesis.

Immunotherapies in CRC are largely disappointing. Tumor vaccines and adoptive T cell transfers have shown little efficacy and immune checkpoint inhibitor therapies are not efficacious in *APC* gene-mutation initiated, microsatellite-stable CRC (14). These immunotherapeutic strategies focus on activating adaptive T cells, and the discouraging outcomes may reflect the inadequate understanding of the unique and complex intestinal immune microenvironment during tumor initiation and progression, especially the importance of innate pathways such as ILC-mediated immunity. Tissue-specific IL-33-activated ILC2s may exert protective anti-tumor immunity in orthotopic, but not subcutaneous, implants of pancreatic cancer (15). Such results indicate the importance of understanding the tissue-specific innate microenvironmental cues that regulate cancer immunity, and how these may be targeted using immunotherapeutics. Here, we identified that CRC patients with high tumor *IL25* expression have reduced survival, and that increased intratumoral ILC2s in CRC patient tissue are associated with impaired anti-tumor immunity. Using a mouse model of APC-mutation-driven CRC, IL-25-activated intratumoral ILC2s promoted intestinal tumorigenesis through enhancing M-MDSC-mediated suppression of anti-tumor immunity. Therapeutic targeting of the pro-tumoral IL-25-ILC2 axis via antibody-mediated blockade reduced intratumoral M-MDSCs and enhanced adaptive T cell IFNγ expression, leading to reduced CRC burden in mice. These results highlight the role of IL-25-activated ILC2s in promoting intestinal tumorigenesis, and suggest that the IL-25-ILC2 axis may be a promising therapeutic target in CRC.

## Results

### IL-25 and ILC2s are associated with human and mouse intestinal tumorigenesis

**Fig. 1:**
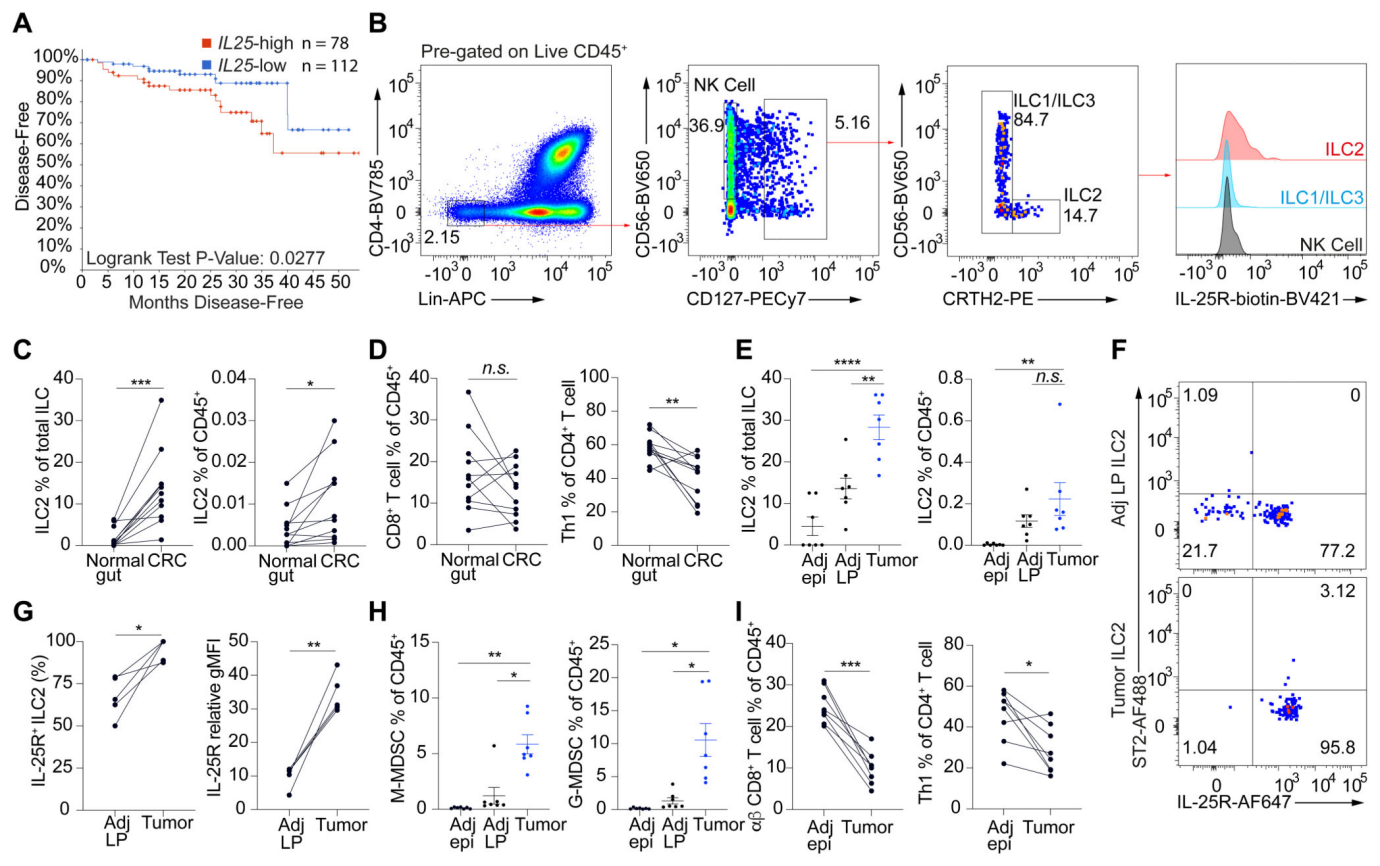
IL-25R expressing ILC2s infiltrate tumors in human and *Apc*^1322T/+^ mouse model of CRC. (**A**) Disease-free survival of CRC patients from the TCGA Firehose Legacy microarray dataset, stratified into two groups by tumor *IL25* expression. *IL25*-high and *IL25*-low groups are defined as patients with CRC that had tumor *IL25* expression above and below the mean of the total samples, respectively. (**B**) Representative FACS plots showing gating strategy and IL-25R expression on ILCs in human CRC samples. (**C** and **D**) Frequency of ILC2s (C), CD8^+^ and Th1 T cells (D) in paired CRC and adjacent normal tissue from CRC patients (*n* = 11). (**E**) Frequency of ILC2s in paired tumors, adjacent normal epithelium (Adj epi) and adjacent lamina propria (Adj LP) in *Apc*^1322T/+^ mice (*n* = 7). (**F**) Representative FACS plots showing IL-25R and ST2 expression in ILC2s from *Apc*^1322T/+^ mouse tumors and Adj LP. (**G**) Expression of IL-25R on ILC2s from paired tumors and Adj LP from *Apc*^1322T/+^ mice (*n* = 5). Relative gMFI, geometric mean fluorescent intensity relative to isotype control. (**H**) Frequency of M-MDSCs and G-MDSCs in tumor, Adj epi, and Adj LP from *Apc*^1322T/+^ mice (*n* = 7). (**I**) Frequency of CD8^+^ T cells and Th1 cells in paired tumor and Adj epi from *Apc*^1322T/+^ mice (*n* = 8). Data pooled from two or more independent experiments and error bars show mean ± SEM. Statistical significance determined by log-rank test (A), one-way ANOVA with Tukey’s post hoc (E and H), and paired two-tailed *t*-test (C, D, G and I). *n.s.* non-signifiacnt, *P < 0.05, **P < 0.01, ***P < 0.001, ****P < 0.0001.

To investigate the potential role of ILC2-activating cytokines and ILC2s in intestinal cancer, we examined human CRC tumor gene expression in large publicly available databases (16–18). CRC patients were stratified into *IL25*-high or *IL25*-low groups based on primary CRC tumor *IL25* gene expression, and their prognosis was compared. There was decreased CRC disease-free (*P* = 0.0277) patient survival in the *Il25*-high group with tumor *IL25* expression above the cohort mean (Fig. 1A and fig. S1, A and B), while there was no difference in tumor size between the two groups (Fig. 1A and fig. S1C). Although there was no statistically significant difference in overall survival (fig. S1A), further subdivision of the *IL25*-high cohort into two groups based on tumor *IL25* expression revealed that the top half showed worse overall survival compared to the lower half (fig. S1D). Conversely, tumor *IL33* gene expression did not associate with differential CRC patient survival when subdivided by cohort mean (fig. S1, E and F), contrary to the positive correlation with survival in pancreatic cancer (15). Fresh, surgically-resected human primary CRC tumors were enriched for IL-25R-positive, Lin^-^CD127^+^CRTH2^+^ ILC2s compared to paired normal colonic tissue (Fig. 1, B and C), consistent with previous studies (19–21). By contrast, CD8^+^ T cells and Th1 (T-bet^+^CD4^+^ T cells) cells did not change in frequency or were reduced, respectively (Fig. 1D and fig. S1G).

Next, we examined ILC2s in the *Apc*^1322T/+^ mouse model of spontaneous intestinal tumorigenesis (22). These mice harbor a mutation closely mirroring APC protein truncations in human CRC (23), resulting in the generation of adenomas which recapitulate early events in human CRC that follows the adenoma-adenocarcinoma sequence (7). Intratumoral ILC2s were confirmed using a combination of immunofluorescence and flow cytometry (fig. S2, A and B). Intestinal ILC2s express KLRG1 but not the IL-33-receptor (ST2) (24), and similar to human CRC, tumors (adenomas) from *Apc*^1322T/+^ mice also harbored increased frequencies of ILC2s (Fig. 1E and fig. S2B), that expressed higher levels of the IL-25R (Fig. 1, F and G). Intestinal tumor ILC2s did not express ST2, confirmed using two different antibody clones (Fig. 1F and fig. S2C). Monocytic and granulocytic MDSCs (M-MDSCs and G-MDSCs respectively), which can suppress anti-tumor T cell immunity (25), were increased in tumors (Fig. 1H and fig. S2D). M-MDSCs in tumors had decreased MHC-II expression (fig. S2E), consistent with a suppressive phenotype (26). Similar to human CRC samples, tumors from *Apc*^1322T/+^ mice had fewer type 1 immune effector cells, including TCRαβ^+^ CD8^+^ T cells and Th1 cells, compared to the adjacent normal gut (Fig. 1I and fig. S2F), while very few NK cells were identified (fig. S2, G and H). γδ T cells also decreased in tumors (fig. S2I), and both tumor-resident CD8^+^ and γδ T cells had decreased granzyme, perforin and IFNγ expression (fig. S2, J and K), suggesting impaired cytotoxic functions. These data showed that IL-25R-expressing ILC2s were preferentially located in the intestinal tumor microenvironment in both mice and humans, and may be associated with the suppressive milieu.

### IL-25 promotes tumor ILC2 infiltration and intestinal tumorigenesis

**Fig. 2:**
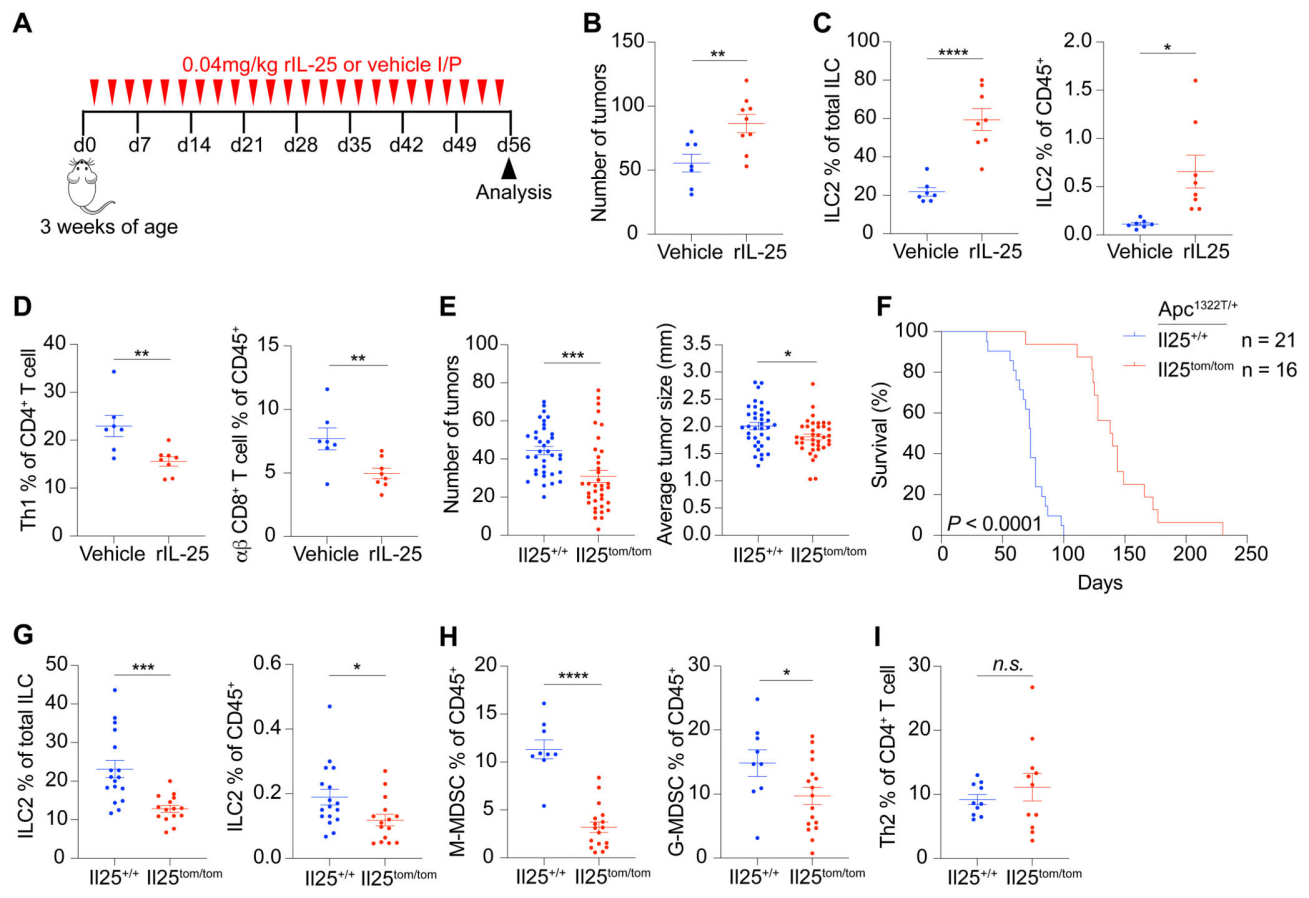
IL-25 promotes intestinal tumors and tumor ILC2s. (**A**) Schematic of rIL-25 or vehicle treatment in *Apc*^1322T/+^ mice, where mice were injected three times per week for eight weeks. (**B**) Number of tumors in vehicle and rIL-25-treated *Apc*^1322T/+^ mice (vehicle, *n* = 7; rIL-25, *n* = 9). (**C**) Tumor ILC2 frequency in vehicle and rIL-25-treated *Apc*^1322T/+^ mice (vehicle, *n* = 7; rIL-25, *n* = 8). (**D**) Frequency of tumor infiltrating Th1 and CD8^+^ T cells in vehicle and rIL-25-treated *Apc*^1322T/+^ mice (vehicle, *n* = 7; rIL-25, *n* = 8). (**E** and **F**) Number of tumors and average tumor size (*Il25*^+/+^, *n* = 36; *Il25*^tom/tom^, *n* = 37) (E) and survival (F) of *Il25*^+/+^ and *Il25*^tom/tom^ *Apc*^1322T/+^ mice. (**G**) Frequency of tumor ILC2s (*Il25*^+/+^, *n* = 17; *Il25*^tom/tom^, *n* = 15) from *Il25*^+/+^ and *Il25*^tom/tom^ *Apc*^1322T/+^ mice. (**H** and **I**) Frequency of M-MDSCs and G-MDSCs (*Il25*^+/+^, *n* = 9; *Il25*^tom/tom^, *n* = 17) (H), and Th2 cells (*Il25*^+/+^, *n* = 10; *Il25*^tom/tom^, *n* = 11) (I), from *Il25*^+/+^ and *Il25*^tom/tom^ *Apc*^1322T/+^ mice. Data pooled from two or more independent experiments and error bars show mean ± SEM. Statistical significance determined by unpaired two-tailed *t*-test, except in (F) by two-sided log-rank test. *n.s.* non-signifiacnt, *P < 0.05, **P < 0.01, ***P < 0.001, ****P < 0.0001.

To investigate the role of IL-25 in intestinal tumorigenesis, *Apc*^1322T/+^ mice were treated with recombinant IL-25 (rIL-25) three times a week for eight weeks (Fig. 2A), which led to a substantial increase in tumor burden (Fig. 2B and fig. S3, A and B). This was not due to tumor epithelial cell proliferation, as there was no difference in Ki67 expression in these cells (fig. S3C). Instead rIL-25 treatment dramatically increased tumor infiltrating ILC2s (Fig. 2C and fig. S3D). There was a decrease in Th1 and CD8^+^ T cells (Fig. 2D and fig. S3E), both known producers of IFNγ, which can inhibit intestinal tumors (27). We found no changes in the frequency of all other immune cell types analyzed (fig. S3, F to M), including MDSCs, macrophages, conventional dendritic cells (cDCs), plasmacytoid dendritic cells (pDCs), mast cells, γδ T cells, Tregs and Th2 cells (Gata3^+^CD4^+^ T cells). These findings indicated that IL-25 preferentially affected innate ILC2s, rather than adaptive Th2 cell frequency, in intestinal tumors.

### Genetic ablation of IL-25 reduces mouse intestinal tumors and increases life-expectancy

We next generated an *Il25*-tomato reporter strain (*Il25*^tom/^+) (fig. S4A), and crossed it with *Apc*^1322T/+^ mice to allow detection of the tomato fluorescent protein as a surrogate for intestinal tumor *Il25* expression. *Il25*-tomato expression was predominantly detected in tumor epithelial cells that co-expressed the tuft cell marker doublecortin-like kinase 1 (DCLK1) (fig. S4B). This was consistent with previous studies that identified tuft cells as the main source of intestinal IL-25 (3), and DCLK1^+^ tuft cells are increased during *Apc*-mutation-mediated intestinal tumorigenesis in mice and human CRC (28, 29). To assess the role of endogenous IL-25, IL-25-deficient (*Il25*^tom/tom^)*Apc*^1322T/+^ mice were generated and compared to IL-25-replete controls. *Il25*^tom/tom^*Apc*^1322T/+^ mice developed fewer and smaller tumors (Fig. 2E), and had increased survival (Fig. 2F and fig. S4C). This was associated with a decrease in tumor ILC2s (Fig. 2G) and M-MDSCs and G-MDSCs (Fig. 2H), while there were no changes in the frequencies of γδ, CD8^+^, Th1 and Th2 T cells, Tregs, macrophages, cDCs, pDCs, mast cells and NK cells (Fig. 2I and fig. S4, D to K). We also investigated the potential roles of IL-33 in regulating intestinal tumor ILC2s. In contrast to IL-25-deficiency, IL-33-deficiency (*Il33*^cit/cit^*Apc*^1322T/+^) (30) had no effect on ILC2 frequency in tumors (fig. S4L), consistent with tumor ILC2 expression of IL-25R, but not ST2 (Fig. 1F and fig. S2C). Instead, confirming previous studies (31, 32), IL-33-deficiency led to a reduction in tumor numbers, and a decrease in tumor mast cells and Tregs, both of which are reported to promote tumorigenesis downstream of IL-33 (fig. S4, M to O). Thus, IL-25 predominantly regulates ILC2s, rather than adaptive Th2 lymphocytes correlating to a tumor-permissive niche.

### ILC2s promote a tumor-permissive microenvironment

**Fig. 3:**
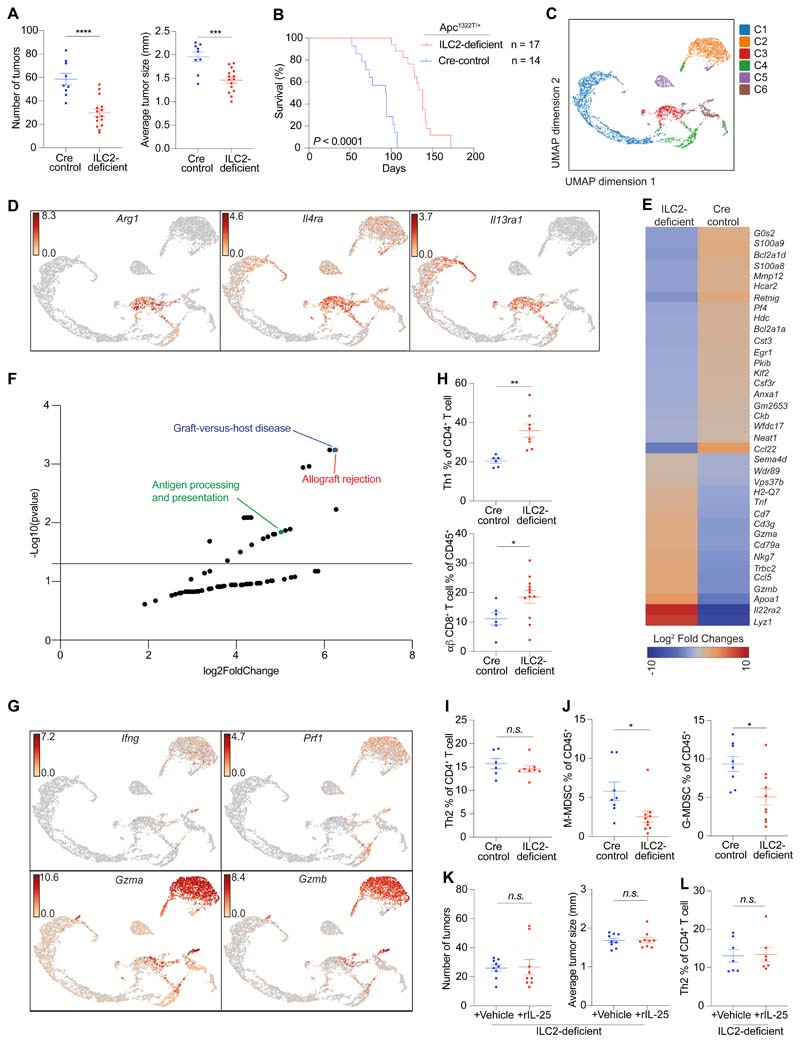
Genetic ablation of ILC2s reduces intestinal tumor burden and increases life-expectancy. (**A** and **B**) Number of tumors and average tumor size (Cre control, *n* = 9; ILC2-deficient, *n* = 15) (A), and survival (B), of ILC2-deficient (*Rora*^f/f^*Il7r*^Cre/+^) and Cre control (*Rora*^+/+^*Il7r*^Cre/+^ or *Rora*^+/f^*Il7r*^Cre/+^) *Apc*^1322T/+^ mice. (**C**) UMAP plot showing combined single-cell RNAseq (scRNAseq) analysis of tumor epithelial cells (C1; CD45^-^EpCAM^+^), M-MDSCs (C3; CD45^+^CD11b^+^Ly6C^+^Ly6G^-^) and total immune population (remaining clusters; CD45^+^) sorted from intestinal tumors from ILC2-deficient and Cre control *Apc*^1322T/+^ mice (>10 tumors pooled from 3 ILC2-deficient mice; 5 tumors pooled from 3 Cre control mice). (**D**) UMAP plot with expression levels (log_2_ expression) of indicated genes (*Arg1, Il4ra* and *Il13ra1*) per individual cell. (**E** and **F**) Heatmap (E) and KEGG pathway analysis (F) of differentially expressed genes identified through scRNAseq, between tumor M-MDSCs from ILC2-deficient and Cre control *Apc*^1322T/+^ mice. (**G**) UMAP plot with expression levels (log2 expression) of indicated genes (*Ifng, Prf1, Gzma* and *Gzmb*) per individual cell. (**H**) Frequency of tumor Th1 cells (Cre control, *n* = 6; ILC2-deficient, *n* = 8) and CD8^+^ T cells (Cre control, *n* = 6; ILC2-deficient, *n* = 12), in ILC2-deficient and Cre control *Apc*^1322T/+^ mice. (**I** and **J**) Frequency of tumor Th2 cells (Cre control, *n* = 6; ILC2-deficient, *n* = 8) (I), and M-MDSCs and G-MDSCs (Cre control, *n* = 8; ILC2-deficient, *n* = 10) (J), in ILC2-deficient and Cre control *Apc*^1322T/+^ mice. (**K**) Number of tumors and average tumor size in vehicle or rIL-25-treated ILC2-deficient (*Rora*^f/f^*Il7r*^Cre/+^) *Apc*^1322T/+^ mice (vehicle, *n* = 9; rIL-25, *n* = 9). (**L**) Frequency of tumor Th2 cells in vehicle or rIL-25-treated ILC2-deficient *Apc*^1322T/+^ mice (vehicle, *n* = 7; rIL-25, *n* = 7). Data pooled from two or more independent experiments and error bars show mean ± SEM (A, B, and H to L). Statistical significance determined by unpaired two-tailed *t*-test, except in (B) by two-sided log-rank test, and in (F) by 10x Genomics Loupe Browser and Enrichr. *n.s.* non-signifiacnt, *P < 0.05, **P < 0.01, ***P < 0.001, ****P < 0.0001.

To determine whether the increased frequency of ILC2s could be responsible for IL-25-dependent intestinal tumorigenesis, we generated ILC2-deficient (*Rora*^f/f^*Il7r*^Cre/+^)*Apc*^1322T/+^ mice (fig. S5A) (4). ILC2-deficient mice had a substantial decrease in tumor numbers and size (Fig. 3A), and a significant (*P* < 0.0001) increase in life-expectancy compared to Cre-control (*Rora*^+/+^*Il7r*^Cre/+^*Apc*^1322T/+^ or *Rora*^+/f^*Il7r*^Cre/+^*Apc*^1322T/+^) mice (Fig. 3B and fig. S5B). RORα may be expressed by subsets of CD4^+^ T cells, and contribute to the suppressive function of skin-resident Tregs during allergic inflammation (33). To address this here, *Rora*^f/f^*Cd4*^Cre/+^*Apc*^1322T/+^ mice were generated. When compared to controls, the frequencies of intratumoral CD4^+^ T cells and Tregs were unaffected (fig. S5C), and there were no differences in tumor numbers and average tumor size (fig. S5D). Altogether, these data demonstrate that similar to IL-25, ILC2s promote *Apc*-mutation-mediated intestinal tumorigenesis.

To understand how ILC2-deficiency may shape the anti-tumoral microenvironment, intestinal tumor epithelial cells (CD45^-^EpCAM^+^) and total immune cells (CD45^+^) were sorted from ILC2-deficient and Cre-control mice, and 10x genomics single-cell RNA sequencing (scRNAseq) was performed. Given the potential association of M-MDSCs with the IL-25-mediated tumor microenvironment (Fig. 2H), we additionally enriched for tumor M-MDSCs for scRNAseq. We observed six clusters (Fig. 3C and fig. S6A) from the tumor samples, including tumor epithelial cells (C1), CD8^+^ T cells (C2), M-MDSCs (C3), plasma B cells (C4), and two B cell clusters which reflected germinal centre light zone (C5) and dark zone (C6) B cells. While an apparent increase in B cells was observed in the ILC2-deficient scRNAseq sample (fig. S6B), upon verification through flow cytometry, there was no difference in B cell frequency between ILC2-deficient and control tumors (fig. S6C). Rather, in both ILC2-deficient and Cre-control *Apc*^1322T/+^ mice, only a small proportion of tumors had B cell infiltration and this did not associate with either genotype (fig. S6, C and D). As ILC2-deficient mice have smaller tumors, more tumors (>10) were pooled to generate the scRNAseq sample compared to the Cre-control (5 tumors), and likely accounted for the apparent increased incidence of B cells observed. Thus, B cells were unlikely to account for the differential tumor burden seen between ILC2-deficient and control mice.

Comparison of tumor epithelial cells (C1) from ILC2-deficient and control tumors identified 56 differentially expressed genes (DEGs), including genes involved in the MHC-I and MHC-II presentation pathways (fig. S6E). KEGG pathway analysis revealed that these were consistent with increased antigen presentation and enhanced immune reactivity in tumor epithelial cells from ILC2-deficient mice (fig. S6F), suggesting the potential increased T cell recognition of tumors in an ILC2-deficient setting and immunosuppressive nature of ILC2s in the tumor microenvironment.

We next looked at the CD45^+^CD11b^+^Ly6C^+^Ly6G^–^ M-MDSC cluster (C3). Tumor M-MDSCs expressed *Arginase 1* (*Arg1*) (Fig. 3D), consistent with a suppressive phenotype (25), and the genes encoding the IL-4Rα and IL-13Rα1 receptor subunits, suggesting their potential ability to respond to ILC2-derived cytokines IL-4 and IL-13. Comparison of M-MDSCs from ILC2-deficient and control tumors revealed a set of DEGs that, similar to tumor epithelial cells, were associated with increased antigen presentation, immune inflammation and reactivity upon KEGG pathway analysis (Fig. 3, E and F). In line with this, M-MDSCs from ILC2-deficient tumors showed a less suppressive phenotype, with reduced expression of *Ccl22, S100a8* and *S100a9* (Fig. 3E). CCL22 is found in a broad range of human cancers with potent pro-tumoral functions (34), while S100A8 and S100A9 are expressed by MDSCs in human CRC patients, are associated with the recruitment and immunosuppressive functions of MDSCs, and correlate with advanced CRC (35). Additionally, ILC2-deficiency led to M-MDSCs downregulating *Bcl2a1a,* which plays key roles in M-MDSC survival (36). The CD8^+^ T cell cluster (C2) expressed *Ifng,* along with genes encoding granzyme A, granzyme B and perforin (Fig. 3G). Direct comparison of CD8^+^ T cells from ILC2-deficient and control tumors revealed very few DEGs (fig. S6G). Due to the limitations on cell numbers and recovery from 10x genomic single-cell RNA sequencing, ILC2s could not be identified in these analyses. Overall, these results suggest that tumor ILC2s may play a role in shaping an immunosuppressive tumor microenvironment and support neoplastic progression, for example by promoting suppressive M-MDSCs.

To further characterize these findings, tumor-infiltrating immune cells from ILC2-deficient and control mice were compared using flow cytometry. ILC2-deficient tumors had increased infiltrating Th1 and CD8^+^ T cells (Fig. 3H and fig. S7A), and enhanced γδ T cell granzyme, perforin and IFNγ expression (fig. S7, B and C), while Th2 cells were unchanged (Fig. 3I and fig. S7D). Immunosuppressive tumor M-MDSCs and G-MDSCs, which can inhibit the anti-tumoral functions of innate γδ and adaptive T cells (25, 37), were reduced (Fig. 3J), while macrophages, cDCs, pDCs, mast cells, NK cells, and Tregs were unchanged (fig. S7, E to J).

Next, to test whether the pro-tumoral effect of exogenous IL-25 was ILC2-dependent, we injected ILC2-deficient *Apc*^1322T/+^ mice with rIL-25 using the same protocol as above (Fig. 2A). rIL-25 did not increase tumor burden (Fig. 3K) or induce ILC2-like cells in ILC2-deficient mice (fig. S8A), indicating that ILC2s are essential for the pro-tumoral effect of IL-25. We still observed a decrease in γδ and CD8^+^ T cells (fig. S8B), however this did not result in increased tumorigenesis in the absence of ILC2s, perhaps because ILC2-deficient mice already displayed highly elevated CD8^+^ T cell tumor infiltration (Fig. 3H). There was no difference in Th1 cells, macrophages, cDCs, pDCs, mast cells, Treg, M-MDSCs, G-MDSCs and Th2 cells (Fig. 3L and fig. S8, C to I). In direct contrast to rIL-25 treatment, rIL-33 treatment increased tumor burden in ILC2-deficient mice, which was associated with increased ST2^+^ tumor Tregs and mast cells (fig. S8, J and K), consistent with previous studies (31, 32). Collectively, our results demonstrate that ILC2s play a non-redundant role in IL-25-mediated intestinal tumorigenesis.

### ILC2s promote M-MDSCs via IL-4 and IL-13 to suppress anti-tumor immunity

**Fig. 4:**
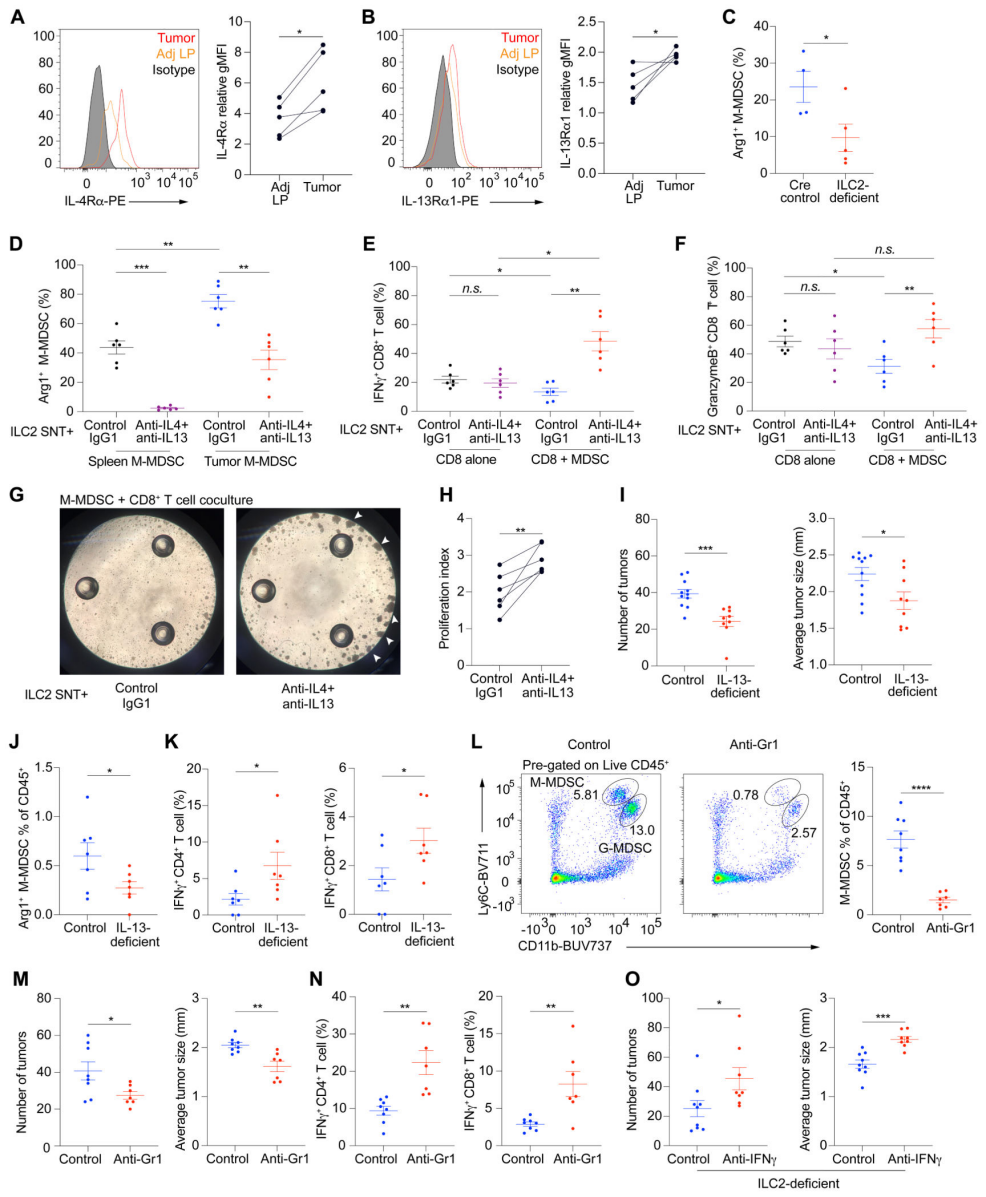
ILC2s promote M-MDSCs via IL-4 and IL-13 to suppress anti-tumor immunity. (**A** and **B**) Representative FACS plots and expression level (relative gMFI) of IL-4Rα (A) and IL-13Rα1 (B), on M-MDSCs in tumor and adjacent lamina propria (Adj LP) from *Apc*^1322T/+^ mice (n = 5). Relative gMFI, geometric mean fluorescent intensity relative to isotype control. (**C**) Arginase 1 (Arg1) expression in tumor M-MDSCs from ILC2-deficient and Cre control *Apc*^1322T/+^ mice (Cre control, *n* = 4; ILC2-deficient, *n* = 5). (**D**) Arg1 expression in splenic and tumor M-MDSCs cultured with ILC2-supernatent (ILC2-SNT) pre-treated with anti-IL-4 and anti-IL-13 neutralizing antibodies (αIL-4/13Ab-ILC2-SNT) or control antibody (conAb-ILC2-SNT) (n = 6). (**E** and **F**) IFNγ (E) and granzyme B (F) expression in CD8^+^ T cells cultured alone or with tumor M-MDSCs, in the presence of αIL-4/13Ab-ILC2-SNT or conAb-ILC2-SNT (n = 6). (**G**) Representative light microscopy images showing tumor M-MDSC and CD8^+^ T cell coculture treated with αIL-4/13Ab-ILC2-SNT or conAb-ILC2-SNT; images taken after 3 days of coculture and arrows point to examples of T cell proliferation foci. (**H**) Quantification of CD8^+^ T cell proliferation when cocultured with tumor M-MDSCs treated with αIL-4/13Ab-ILC2-SNT or conAb-ILC2-SNT (n = 6). (**I**) Number of tumors and average tumor size in control (*Il13*^+/+^ and *Il13*^+/tom^) and IL-13-deficient (*Il13*^tom/tom^) *Apc*^1322T/+^ mice (control, *n* = 11; IL-13-deficient, *n* = 9). (**J**) Frequency of Arg1 expressing M-MDSCs in control and IL-13-deficient *Apc*^1322T/+^ mice (control, *n* = 7; IL-13-deficient, *n* = 7). (**K**) IFNγ expression in tumor CD4^+^ and CD8^+^ T cells, from control and IL-13-deficient *Apc*^1322T/+^ mice (control, *n* = 7; IL-13-deficient, *n* = 7). (**L**) Representative FACS plots and frequency of M-MDSCs in control rIgG2b and anti-Gr1 treated *Apc*^1322T/+^ mice (control, *n* = 8; treatment, *n* = 7). (**M**) Number of tumors and average tumor size in control and anti-Gr1 treated *Apc*^1322T/+^ mice (control, *n* = 8; treatment, *n* = 7). (**N**) IFNγ expression in tumor CD4^+^ and CD8^+^ T cells, from control and anti-Gr1 treated *Apc*^1322T/+^ mice (control, *n* = 8; treatment, *n* = 7). (**O**) Number of tumors and average tumor size in control rIgG1 or anti-IFNγ treated ILC2-deficient (*Rora*^f/f^*Il7r*^Cre/+^) *Apc*^1322T/+^ mice (control, *n* = 9; anti-IFNγ, *n* = 8). Data pooled from 25 two or more independent experiments and error bars show mean ± SEM. Statistical significance determined by paired two-tailed *t*-test (A, B, and H), unpaired two-tailed *t*-test (C, and I to O), and one-way ANOVA with Tukey’s post hoc (D to F). *n.s.* non-signifiacnt, *P < 0.05, **P < 0.01, ***P < 0.001, ****P < 0.0001.

Given the association of the pro-tumoral IL-25-ILC2 axis with MDSCs, and the expression of *Il4ra* and *Il13ra1* by M-MDSCs, we sought to investigate whether ILC2-derived IL-4 and IL-13 promote MDSC function to suppress anti-tumor immunity. Flow cytometry analysis showed that M-MDSCs from intestinal tumors upregulated the IL-4Rα and IL-13Rα1 receptor subunits as compared to the few M-MDSCs in the normal gut (Fig. 4, A and B), while G-MDSCs expressed IL-4Rα but not IL-13Rα1 (fig. S9, A and B), indicating that MDSCs may respond to IL-4 and IL-13. Furthermore, tumor M-MDSCs from ILC2-deficient mice had decreased Arg1 expression as compared to controls (Fig. 4C and fig. S9C), and Arg1 plays a role in MDSC-mediated T cell suppression (25). We next collected IL-4 and IL-13-containing supernatants from cultured primary intestinal ILC2s (ILC2-SNT) (fig. S9D) and treated them with either a combination of anti-IL-4 and anti-IL-13 neutralizing antibodies (αIL-4/13Ab-ILC2-SNT), or isotype control antibody (conAb-ILC2-SNT), before adding them to purified intestinal tumor M-MDSCs or splenic M-MDSCs from *Apc*^1322T/+^ mice.

Following culture, both splenic and tumor M-MDSCs treated with αIL-4/13Ab-ILC2-SNT had decreased Arg1 expression compared to conAb-ILC2-SNT-treated M-MDSCs (Fig. 4D), indicating that intestinal ILC2-derived IL-4 and IL-13 can promote Arg1 expression in M-MDSCs.

Next, we investigated whether ILC2-derived IL-4 and IL-13 could affect M-MDSC-mediated CD8^+^ T cell suppression. Purified tumor M-MDSCs were pre-treated with αIL-4/13Ab-ILC2-SNT or conAb-ILC2-SNT, before coculture with CD8^+^ T cells. As controls, CD8^+^ T cells alone were cultured with αIL-4/13Ab-ILC2-SNT or conAb-ILC2-SNT. Tumor M-MDSCs treated with conAb-ILC2-SNT suppressed CD8^+^ T cell IFNγ and granzyme B production (Fig. 4, E and F and fig. S9E). Neutralizing IL-4 and IL-13 in the ILC2-SNT released the suppression on IFNγ and granzyme B production by CD8^+^ T cells cocultured with M-MDSCs, while having no direct effect on CD8^+^ T cells cultured alone. Similarly, CD8^+^ T cells from αIL-4/13Ab-ILC2-SNT-treated M-MDSC-CD8^+^ T cell cocultures showed increased TNFα and perforin expression compared to conAb-ILC2-SNT-treated cocultures (fig. S9F), and increased cell proliferation (Fig. 4, G and H, and fig. S9G), demonstrating that ILC2-derived IL-4 and IL-13 promoted the suppressive functions of tumor M-MDSCs. Finally, CD8^+^ T cells from M-MDSC-CD8^+^ T cell cocultures treated with αIL-4/13Ab-ILC2-SNT, showed increased expression of IFNγ and perforin compared to CD8^+^ T cells cultured with αIL-4/13Ab-ILC2-SNT alone (Fig. 4E and fig. S9F), indicating that reprogrammed M-MDSCs promoted CD8^+^ T cell function when ILC2-derived IL-4 and IL-13 was blocked.

Analysis of cytokine production by intestinal tumor-derived immune cells *ex vivo,* revealed that tumor ILC2s expressed IL-4 (fig. S10A), and rather than tumor CD4^+^ T cells, are the predominant source of IL-13 in the tumor microenvironment (fig. S10, B and C). This suggested an innate source of these cytokines, and the persisting role of ILC2-mediated innate signals in chronic tumor settings. Next, we tested the impact of IL-13-ablation on intestinal tumorigenesis *in vivo.* IL-13-deficiency (*Il13*^tom/tom^*Apc*^1322T/+^) (38) decreased tumor burden (Fig. 4I), and reduced tumor Arg1^+^ M-MDSCs (Fig. 4J) while increasing IFNγ expression in tumor-infiltrating CD4^+^ and CD8^+^ T cells (Fig. 4K and fig. S10D). The reduced tumor burden in IL-13-deficient mice was unlikely to be due to direct effects on tumor epithelial cells, as they lacked IL-13Rα1 protein expression (fig. S10E), in contrast to tumor M-MDSCs (Fig. 4B). Finally, the pro-tumoral roles of MDSCs were confirmed using anti-GR1-depleting antibody treatment, which significantly reduced tumor MDSCs (Fig. 4L and fig. S11A), leading to reduced tumor burden (Fig. 4M) and increased IFNγ expression in CD4^+^ and CD8^+^ T cells (Fig. 4N and fig. S11B), consistent with an enhanced anti-tumor immune microenvironment.

Given the association of type 1 immune cells, including Th1 and CD8^+^ T cells, and IFNγ expression with the protective response observed in IL-13-deficient and ILC2-deficient mice, we assessed the role of CD8^+^ T cells and IFNγ in the enhanced anti-tumor immune response observed in the absence of ILC2s. Antibody-mediated neutralization of IFNγ in ILC2-deficient *Apc*^1322T/+^ mice resulted in an increase in the number and size of tumors (Fig. 4O), revealing that increased IFNγ production contributed to the decreased tumor burden observed in ILC2-deficient mice. Conversely, antibody-mediated depletion of CD8^+^ cells did not increase tumor burden in ILC2-deficient mice (fig. S11C), suggesting that there are additional IFNγ-producing immune cell populations such as Th1 and γδ T cells that contribute to the reduced tumor burden observed in ILC2-deficient mice. Together, these data suggest that IL-4 and IL-13 produced by ILC2s in the tumor niche promote M-MDSC-mediated suppression of anti-tumor immunity and IFNγ production, demonstrating the role of sustained innate signals in shaping the tumor microenvironment.

### Mechanistic pathway conservation in human CRC

**Fig. 5:**
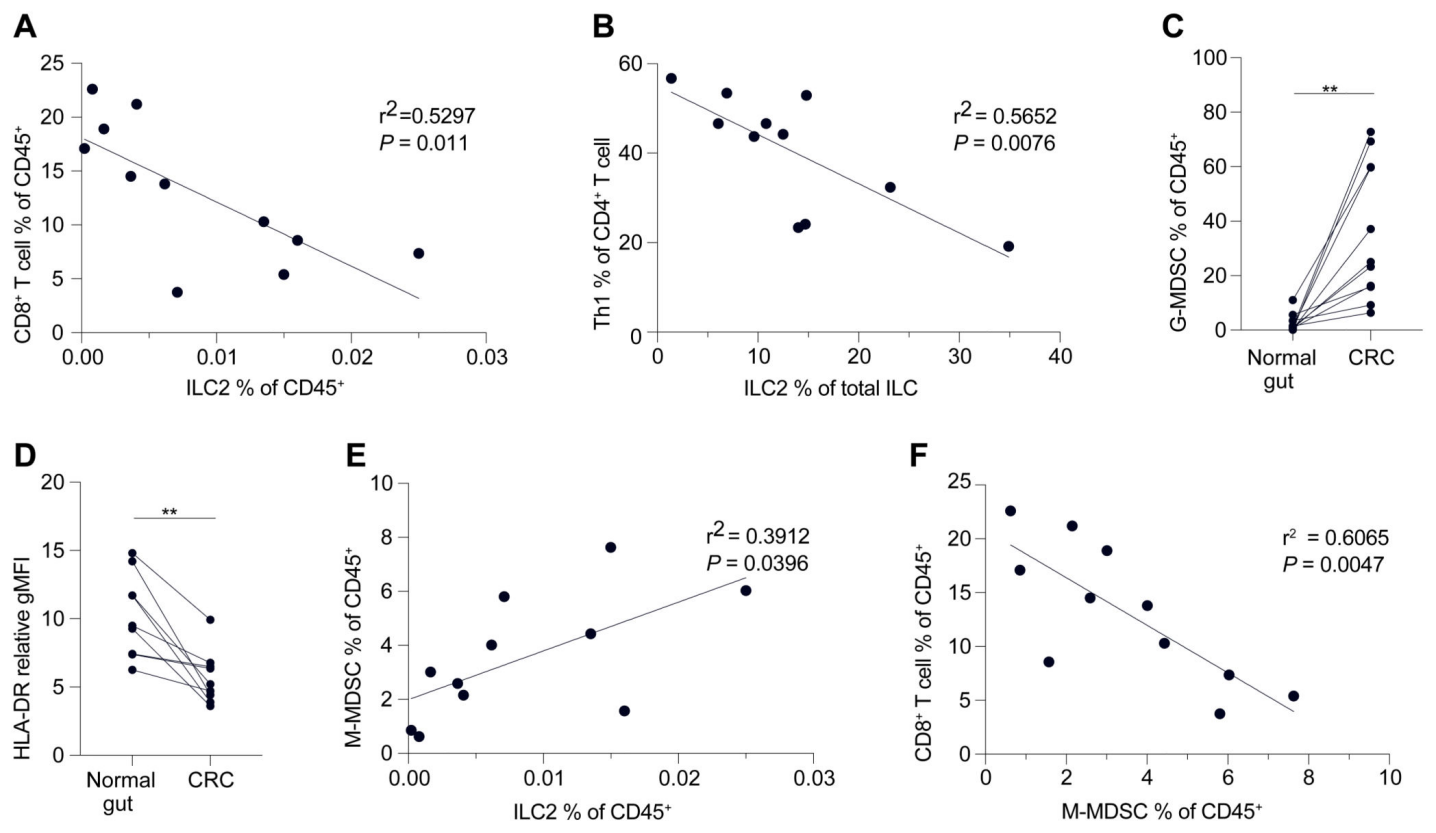
ILC2s are associated with impaired anti-tumor immunity in human CRC. (**A** and **B**) Correlation of CD8^+^ T cells and ILC2s (A), and Th1 cells and ILC2s (B), in human CRC samples. (**C**) Frequency of G-MDSCs in human CRC and adjacent normal gut. (**D**) HLA-DR expression level (relative gMFI) of M-MDSCs in human CRC and adjacent normal gut. Relative gMFI, geometric mean fluorescent intensity relative to FMO control. FMO, full-minus-one. (**E**) Correlation of M-MDSCs and ILC2s in human CRC samples. (**F**) Correlation of M-MDSCs and CD8^+^ T cells in human CRC samples. Each dot or pair of dots indicates an individual human CRC patient; (A to C, E and F) *n* = 11; (D), *n* = 9. Data pooled from two or more independent experiments and error bars show mean ± SEM. Statistical significance determined by paired two-tailed *t*-test (C and D), and by Pearson’s rank correlation coefficient (A, B, E and F). **P < 0.01.

Our results demonstrated that the innate IL-25-ILC2 axis supports intestinal tumor progression in part through promoting MDSCs and suppressing anti-tumoral T cells and IFNγ. To investigate this in humans, we analyzed immune cells from fresh human CRC samples. Th1 and CD8^+^ T cell infiltration is amongst the strongest positive prognostic factors for survival of human CRC patients across all stages of disease (39, 40). Correlation analysis showed a significant negative correlation between tumor CD8^+^ T cells and ILC2s in human CRC (Fig. 5A), and between Th1 cells and ILC2s (Fig. 5B), suggesting that CRCs with a higher ILC2 frequency may have impaired anti-tumor T cell responses and worse prognosis. Similar to mice, human CRCs had increased CD11b^+^CD14^-^CD15^+^CD33^int^ G-MDSCs (Fig. 5C and fig. S12A), and CD11b^+^CD14^+^CD15^-^CD33^high^HLA-DR^low/-^ M-MDSCs had decreased HLA-DR compared to the adjacent normal gut (Fig. 5D). Tumor M-MDSCs positively correlated with ILC2s in CRC patients (Fig. 5E), suggesting that human M-MDSC recruitment in CRC may mirror the ILC2-dependent requirement for M-MDSC maintenance observed in mouse intestinal tumors. Finally, tumor infiltrating CD8^+^ T cells had an exhausted phenotype with decreased T-bet and increased inhibitory checkpoint receptor expression (fig. S12, B to D), and negatively correlated with M-MDSCs in human CRC (Fig. 5F), consistent with the ability of M-MDSCs to suppress CD8^+^ T cells. The similar immune cell representation in human CRC and *Apc*^1322T/+^ mouse intestinal tumors suggested potential mechanistic conservation in which the IL-25-ILC2 axis associated with impaired survival.

### Therapeutic blockade of the IL-25-ILC2 axis promotes anti-tumor immunity and decreases tumor burden

**Fig. 6:**
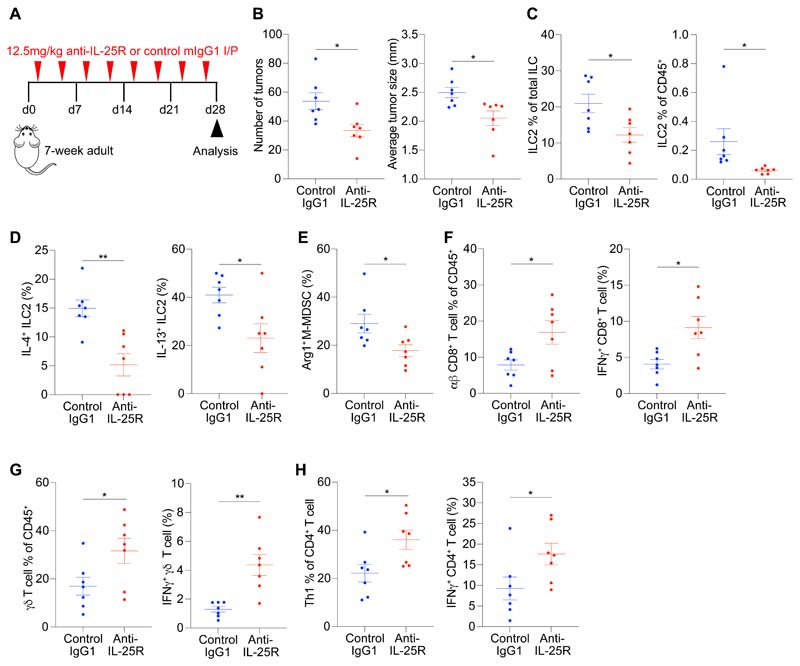
Therapeutic intervention blocking the IL-25-ILC2 axis promotes anti-tumor immunity and decreases tumor burden. (**A**) Schematic of anti-IL-25R or control treatment starting in adult *Apc*^1322T/+^ mice with established tumors; mice were injected two times per week for four weeks. (**B**) Number of tumors and average tumor size in anti-IL-25R or control IgG1-treated *Apc*^1322T/+^ mice (control IgG1, *n* = 7; anti-IL-25R, *n* = 7). (**C** and **D**) Frequency (C), and IL-4 and IL-13 expression (D) in tumor ILC2s from anti-IL-25R or control IgG1-treated *Apc*^1322T/+^ mice (control IgG1, *n* = 7; anti-IL-25R, *n* = 7). (**E**) Arginase 1 (Arg1) expression in tumor M-MDSCs from anti-IL-25R or control IgG1-treated *Apc*^1322T/+^ mice (control IgG1, *n* = 7; anti-IL-25R, *n* = 7). (**F** to **H**) Frequency and IFNγ expression in tumor CD8^+^ T cells (F), *gd* T cells (G), and Th1 cells (H), from anti-IL-25R or control IgG1-treated *Apc*^1322T/+^ mice (control IgG1, *n* = 7; anti-IL-25R, *n* = 7). Data collected from age-matched female mice treated with anti-IL-25R or control IgG1, and pooled from two independent experiments; error bars show mean ± SEM. Statistical significance determined by unpaired two-tailed *t*-test. *P < 0.05, **P < 0.01.

We assessed whether the innate IL-25-ILC2 immune axis could be targeted therapeutically using an anti-IL-25R (clone D9.2) blocking antibody in adult *Apc*^1322T/+^ mice with established tumors (Fig. 6A) (22). Anti-IL-25R treatment significantly reduced tumor number and size (Fig. 6B), and decreased IL-4 and IL-13 expressing tumor ILC2s (Fig. 6, C and D). There were also fewer Arg1-expressing tumor M-MDSCs in anti-IL-25R-treated mice (Fig. 6E and fig. S13A). Indeed, anti-IL-25R-treated adult mice had increased CD8^+^ T cell, γδ T cell and Th1 cell frequencies and elevated IFNγ production in tumors (Fig. 6, F to H, and fig. S13, B to E), signifying a switch to anti-tumor immunity. Additionally, prophylactic anti-IL-25R treatment in ~3-week-old young mice, before the appearance of tumors, (fig. S14A) led to a similar decrease in tumor burden (fig. S14B) and ILC2s (fig. S14C). Tumor MDSCs also decreased (fig. S14D), along with a concomitant increase in CD8^+^ T cells (fig. S14E). Th1 and Th2 cells, macrophages, cDCs, pDCs, mast cells, γδ T cells and Tregs did not change in frequency (fig. S14, F to M).

**Fig. 7:**
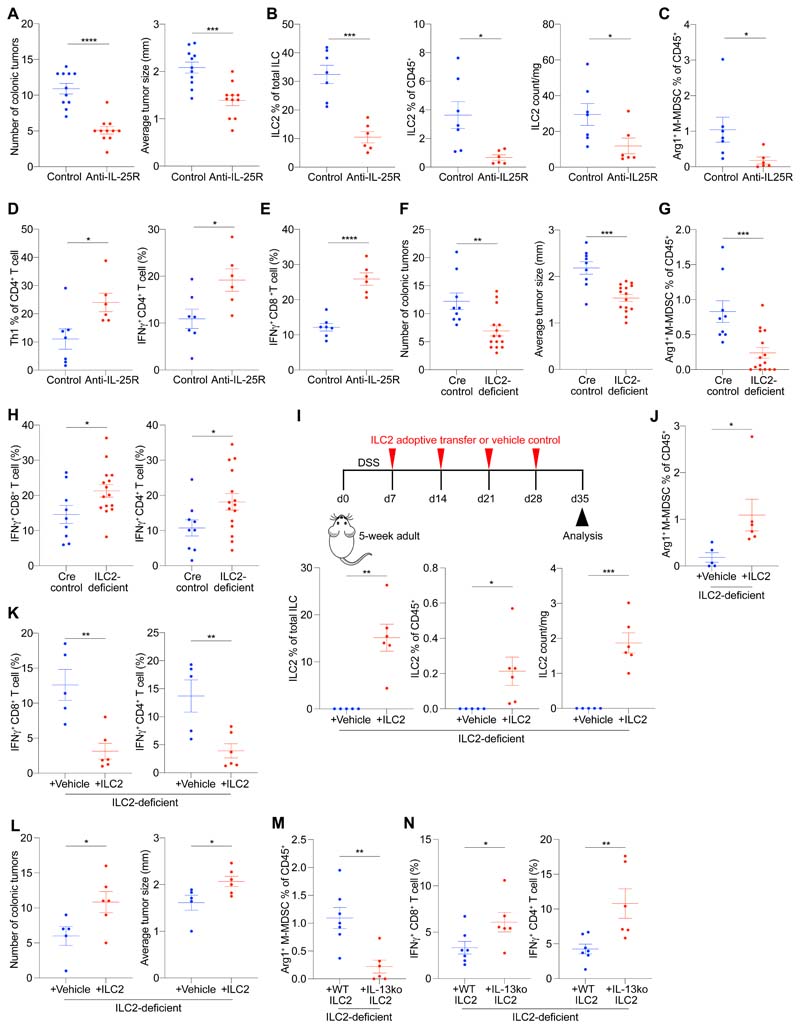
Therapeutic anti-IL-25R treatment shows mechanistic conservation against mice colonic adenocarcinoma. (**A**) Number of colonic tumors and average tumor size in anti-IL-25R or control IgG1-treated *Apc*^1322T/+^-DSS mice (control IgG1, *n* = 11; anti-IL-25R, *n* = 11). *Apc*^1322T/+^-DSS mice, dextran sulfate sodium treated *Apc*^1322T/+^ mice. (**B** and **C**) Frequency of ILC2s (B) and Arg1+ M-MDSCs (C) in anti-IL-25R or control IgG1-treated Apc^1322T/+^-DSS mice (control IgG1, *n* = 7; anti-IL-25R, *n* = 6). (**D**) Frequency of tumor Th1 cells and CD4^+^ T cell IFNγ expression in anti-IL-25R or control IgG1-treated *Apc*^1322T/+^-DSS mice (control IgG1, *n* = 7; anti-IL-25R, *n* = 6). (**E**) IFNγ expression in tumor CD8^+^ T cells from anti-IL-25R or control IgG1-treated *Apc*^1322T/+^-DSS mice (control IgG1, *n* = 7; anti-IL-25R, *n* = 6). (**F**) Number of colonic tumors and average tumor size (Cre control, *n* = 9; ILC2-deficient, *n* = 15) in ILC2-deficient (*Rora*^f/f^*Il7r*^Cre/+^) and Cre control (*Rora*^+/+^*Il7r*^Cre/+^) *Apc*^1322T/+^-DSS mice. (**G**) Frequency of colonic tumor Arg1^+^ M-MDSCs (Cre control, *n* = 9; ILC2-deficient, *n* = 15) from ILC2-deficient and Cre control *Apc*^1322T/+^-DSS mice. (**H**) IFNγ expression in colonic tumor CD8^+^ and CD4^+^ T cells (Cre control, *n* = 9; ILC2-deficient, *n* = 15) from ILC2-deficient and Cre control *Apc*^1322T/+^-DSS mice. (**I**) Schematic (top) of ILC2-deficient *Apc*^1322T/+^-DSS mice adoptively transferred with ILC2s or control, and frequency (bottom) of colonic tumor ILC2s at point of analysis (Control, *n* = 5; ILC2, *n* = 6). (**J**) Frequency of Arg1^+^ M-MDSCs in ILC2-deficient *Apc*^1322T+^-DSS mice adoptively transferred with ILC2s or control (Control, *n* = 5; ILC2, *n* = 6). (**K** and **L**) IFNγ expression in colonic tumor CD8^+^ and CD4^+^ T cells (K), and colon tumor number and size (L), in ILC2-deficient *Apc*^1322T/+^-DSS mice adoptively transferred with ILC2s or control (Control, *n* = 5; ILC2, *n* = 6). (**M** and **N**) Frequency of colonic tumor Arg1^+^ M-MDSCs (M), and CD8^+^ and CD4^+^ T cell IFNγ expression (N) in ILC2-deficient *Apc*^1322T/+^-DSS mice adoptively transferred with wild type or IL-13-deficient ILC2s (wild type, *n* = 7; IL-13-deficient, *n* = 6). Data collected from age-matched female mice treated with DSS, and pooled from two or more independent experiments; error bars show mean ± SEM. Statistical significance determined by unpaired two-tailed *t*-test. *P < 0.05, **P < 0.01, ***P < 0.001, ****P < 0.0001.

Having established the role of the IL-25-ILC2-M-MDSC axis during adenoma development in the small intestine, we investigated its role during the progression to invasive adenocarcinoma in the colon using a model more closely mirroring human CRC. We used *Apc*-mutant mice treated with dextran sulfate sodium (DSS), which induces robust *Apc*-mutation-dependent invasive colonic adenocarcinoma (41). *Apc*^1322T/+^ mice were fed DSS for induction of colonic adenocarcinoma before anti-IL-25R treatment. Anti-IL-25R treatment robustly decreased colonic tumor burden (Fig. 7A and fig. S15A), and led to a decrease in tumor ILC2s, Arg1^+^ M-MDSCs and G-MDSCs (Fig. 7, B and C, and fig. S15, B and C). Th1 cells and IFNγ-producting CD4^+^ and CD8^+^ T cells were also increased (Fig. 7, D and E, and fig. S15, D and E), consistent with improved anti-tumor immunity upon anti-IL-25R treatment.

Comparing ILC2-deficient to control *Apc*^1322T/+^ mice treated with DSS, ILC2-deficiency led to reduced colonic tumor burden (Fig. 7F and fig. S15F), a similar reduction in tumor G-MDSCs and Arg1^+^ M-MDSCs (Fig. 7G and fig. S15, G and H), and an increase in IFNγ-expressing CD4^+^ and CD8^+^ T cells (Fig. 7H and fig. S15I). Adoptive transfer of IL-25-activated ILC2s into DSS-treated ILC2-deficient *Apc*^1322T/+^ mice (Fig. 7I) resulted in increased Arg1^+^ M-MDSCs (Fig. 7J and fig. S16A), reduced IFNγ-expressing intratumoral CD4^+^ and CD8^+^ T cells (Fig. 7K and fig. S16B), and an elevated tumor burden (Fig. 7L and fig. S16C). Finally, adoptive transfer of wild type or IL-13-deficient ILC2s into DSS-treated ILC2-deficient Apc^1322T^+ mice confirmed that ILC2-derived IL-13 was critical in promoting colonic tumor Arg1^+^ M-MDSCs (Fig. 7M and fig. S16D) and suppressing CD4^+^ and CD8^+^ T cell IFNγ expression (Fig. 7N and fig. S16E). Thus, anti-IL-25R treatment has therapeutic efficacy against colonic adenocarcinoma by shifting the intestinal immune response from a suppressive ILC2-dependent innate-immune response towards active anti-tumoral immunity.

## Discussion

Here, we found that sustained innate IL-25 and ILC2 signals helped to maintain a cancer-permissive microenvironment in intestinal polyps and colorectal adenocarcinoma, by preventing anti-tumoral T cell and IFNγ-mediated immunity. In human CRC patients, Th1 and CD8^+^ T cell infiltration is amongst the strongest positive prognostic factors for survival across all stages of disease (39, 40). We found that IL-25R^+^ ILC2s are enriched in human CRCs, positively correlated with M-MDSCs, while negatively correlating with anti-tumor Th1 and CD8^+^ T cells. Our findings confirm and extend studies that found an abundance of ILC2s in human CRC (19–21), and demonstrate that the increased ILC2s may be associated with impaired anti-tumor T cell immunity, directly contrasting with the protective role of MHC-II^+^ ILC3s in CRC (42). Further, our analyses identified that high CRC *IL25* expression is associated with poor overall and disease-free survival in CRC patients (16–18), supporting the role of the innate IL-25-ILC2-M-MDSC axis in promoting intestinal tumorigenesis and the potential for therapeutic intervention.

Most immunotherapies tested in human CRC have directly targeted adaptive immune responses. Here, we identified that the innate IL-25-ILC2-MDSC immune axis promotes CRC, and can be targeted therapeutically. The use of the (*Rora*^f/f^*Il7r*^Cre/+^)*Apc*^1322T/+^ ILC2-deficient model allowed preferential deletion of ILC2s while having minimal effects on adaptive Th2 cells (43), attributing the pro-tumoral effect to innate type 2 signals. Deletion of RORα in adaptive T cells did not affect intestinal tumor burden, and exogenous IL-25 treatment in ILC2-replete and ILC2-deplete mice showed that ILC2s are essential for IL-25-mediated intestinal tumorigenesis. Adoptive transfer experiments further confirmed that ILC2-derived IL-13 promotes immunosuppressive tumor M-MDSCs and reduces IFNγ^+^ T cells. Interestingly, human CRC have reduced MHC expression (44), and defects in tumor cell antigen presentation can facilitate cancer immune escape from T cell-mediated tumor rejection (45), and promote invasion and metastasis (46). Our study revealed that tumor epithelial cells and M-MDSCs from ILC2-deficient *Apc*^1322T/+^ mice showed enhanced expression of genes encoding MHC and antigen presentation pathways upon ILC2-deficiency. Along with the marked increase in tumor-infiltrating Th1 and CD8^+^ T cells, these observations indicate that ILC2-depletion may improve tumor antigen presentation and anti-tumor immunity, and is consistent with the reduced tumor burden.

Our data demonstrate that tissue specialization of IL-25-responsive tumor-promoting intestinal ILC2s have opposite functions to the tissue-specific IL-33-activated ILC2s that are protective in orthotopic pancreatic cancer and melanoma (15, 47). Previous studies attributed the pro-tumoral effect of IL-33 during *Apc*-mutation-mediated intestinal tumorigenesis to ST2^+^ Tregs and mast cells (31, 32). Accordingly, we found that IL-33-deficiency led to reduced intestinal tumor Tregs and mast cells, while not affecting ILC2 frequency. Conversely, modulation of IL-25-signaling affected intestinal tumor ILC2s, while mast cells and Tregs were unaffected. Intestinal ILC2s express the highest levels of IL-25R compared to those from any other tissue, while ST2 expression is minimal (24). We found that intestinal tumor ILC2s had increased expression of IL-25R, but did not express ST2, and while the pro-tumoral effects of IL-25 required ILC2s, IL-33 acted independently of ILC2s correlating instead with mast cell and Treg expansion. Unlike in many other types of cancers such as melanoma, cervical, renal, hepatocellular and breast cancer where Tregs are pro-tumoral and associated with poor patient prognosis, recent studies in both mice and human indicate that Tregs are protective against APC-mutation-mediated intestinal tumorigenesis (48, 49). While we cannot completely rule out a potential change in Treg function or contribution of Tregs downstream IL-25 during *Apc*-mutation-mediated tumorigenesis, the lack of change in Treg frequency, along with the anti-tumorigenic role, suggest that Tregs are unlikely to act downstream pro-tumorigenic IL-25 signals. Conversely, ablation of ILC2s or MDSCs decreased intestinal tumorigenesis. These data suggest that the innate IL-25-ILC2 axis is a separate pathway previously unappreciated in promoting intestinal tumorigenesis. By contrast, lung ILC2s show a largely opposite receptor profile compared to intestinal ILC2s, predominantly expressing ST2 but not IL-25R (24), and can either promote or suppress cancer metastasis to the lung in response to IL-33 (50, 51). While IL-25-deficiency led to a decrease in both intestinal tumor number and size, IL-33-deficiency in our study only reduced tumor number and not tumor size, in keeping with them mediating separate pro-tumorigenic pathways. As we have shown in human CRC patients, high tumor *IL25* expression is associated with poor CRC patient survival, whereas *IL33* expression does not discriminate between better or worse prognosis. These data suggest that the IL-25-ILC2 axis may have a greater impact on tumor progression in human CRC.

Our data highlight how the ontology of intestinal tumors can lead to highly disparate immune reactions. Indeed, inhibiting IL-25-signalling or ILC2s in an AOM/DSS model of colitis-associated-cancer (CAC) subtype of CRC exacerbated tumors (52–54), while subcutaneous combined implants of sorted human ILC2s and EpCAM^+^ CRC tumor cells in immunodeficient mice can promote tumor growth, albeit in an immunocompromised setting (19). The differences are likely to be due to the distinct models used, which characterize different subtypes of CRC. The AOM/DSS model of CAC harbors distinct genetic changes compared to conventional sporadic CRC that follows the adenoma-adenocarcinoma sequence, and lacks mutations in APC (55). In humans, 13% of CAC have mutations in *APC,* compared to 80% in sporadic CRC, and immune-profiling found distinct immune signatures between sporadic CRC and CAC that are consistent with different disease phenotypes and aetiologies (56, 57). Accordingly, experimental ablation of Tregs in AOM/DSS-mediated CAC reduced tumor burden (58), while Treg depletion in *Apc*^min/+^ mice increased tumorigenesis (48). As CAC accounts for a small proportion of total CRC (1-2%) (56), Tregs are associated with improved survival in human CRC overall in a large systematic review and meta-analysis compiling eight studies (49). Similarly, elevated *Mmp9* (encoding metalloproteinase-9, MMP9) expression is widely associated with increased CRC cell proliferation, invasion, metastasis and reduced patient survival (59). While genetic deficiency of MMP9 decreased *Apc*-mutation-mediated intestinal tumorigenesis in mice (60), deletion of MMP9 in AOM/DSS-treated mice instead enhanced CAC development (61), suggesting that while MMP9 has oncogenic roles in *Apc*-mutation-mediated sporadic CRC, it may elicit tumor suppressive effects during CAC. IL-25-deficiency reduced *Mmp9* expression in the AOM/DSS model of CAC (52), which may contribute to the opposing effects of IL-25 observed in different subtypes of CRC. These observations highlight that the distinct genetic mutations and immune environment of different CRC subtypes, may potently influence disease development and application of therapeutics.

Using the *Apc*-mutant-DSS model of colonic adenocarcinoma (41), our results show that the type 2 immune microenvironment driven by the IL-25-ILC2 axis promotes *Apc*-mutation-dependent CRC, and therapeutic anti-IL-25R treatment reduced CRC burden. Supporting the pro-tumoral role of type 2 immunity, early epidemiological studies associated intestinal helminth *Schistosoma japonicum* infections with increased CRC risk and incidence, accounting for up to 24% of CRC in endemic areas (62), and correlated with increased metastasis and reduced survival (63). As the IL-25-ILC2s axis has well-established roles in protecting against intestinal helminth infections (1, 3), our findings suggest that the protective IL-25-ILC2 response during chronic helminth infections may concomitantly predispose individuals to sporadic CRC development. Overall, the relative roles of the epithelium-derived cytokines IL-25 and IL-33, and the involvement of ILC2s in tumorigenesis, should not be generalized. Therapeutic blockade of the IL-25-ILC2 axis may be beneficial in CRC patients where *APC*-mutation predominates (80% of all CRC) and should be explored, but possibly not in the rarer and mutationally distinct non-*APC*-mutation-initiated CRCs and CAC subtypes where IL-25 may instead suppress tumorigenesis (56). Furthermore, our work focused on autochthonous models and sets the foundation for future studies to explore the involvement of the IL-25-ILC2 axis in models of more clinically advanced stages of CRC, such as orthotopic organoid transplant (13).

Although MDSCs suppress anti-tumor immunity, targeting them therapeutically has proven challenging (64, 65, 66, 67) ILC2s promoted the suppressive capacities of tumor M-MDSCs, and *in vitro* blockade of ILC2-derived IL-4 and IL-13 reprogrammed M-MDSCs to adopt an immunostimulatory phenotype, promoting IFNγ expression in CD8 T cells. This is consistent with the reduced expression of many of the known genes that contribute to M-MDSC-suppressive function as identified in our scRNAseq dataset, including *S100a8, S100a9, Ccl22* and *Bcl2a1a* which decreased in tumor M-MDSCs in ILC2-deficient settings (34–36). Experimental IL-25-signaling blockade (using the D9.2 clone which also blocks human IL-25R) inhibited ILC2 production of IL-4 and IL-13, limiting the suppressive capacities of MDSCs, thereby permitting the liberation of anti-tumor T cell and IFNγ-mediated immunity and reduced CRC. Thus, blocking IL-25-signaling in human *APC*-mutation-mediated CRC may be promising therapeutically. In particular, this could be combined with the use of *IL25* as a biomarker to identify patients with poor prognosis who co-present with high CRC *IL25* gene expression. IL-25-responsive inflammatory ILC2s appear to originate from the gut and migrate from the intestines to the lungs and actuate anti-helminth immunity, serving as an important source of IL-4 and IL-13 (68). Whether cancers at other sites of the body may recruit these pro-tumorigenic IL-25-responsive intestinal ILC2s potentially to sustain MDSCs remains to be explored. As anti-IL-25R treatment shifts the balance away from suppressive MDSCs and towards anti-tumoral IFNγ^+^ Th1 and CD8^+^ T cell infiltration, targeting IL-25-responsive ILC2s might have clinical benefit in CRC patients.

## Materials and Methods

### Study Design

The objective of this study was to characterise how IL-25, IL-33 and ILC2s contribute to the immune responses that arise during APC-mutation-mediated adenoma and adenocarcinoma. A combination of genetic mouse models and human CRC samples were employed in this study, and a variety of strategies were used including genetic and pharmacological manipulation, *in vitro* culture systems, immunostaining, biochemical analysis and scRNAseq. Genetic IL-25-deficient mice, and rIL-25 treatment experiments were performed complimentarily to assess the role of IL-25 during intestinal tumorigenesis. Similarly, genetic ILC2-deficiency and ILC2 adoptive transfer experiments were performed to investigate ILC2 function during intestinal tumor development. Finally, anti-IL-25R treatment experiments were performed to assess therapeutic efficacy of IL-25-ILC2 axis blockade against CRC.

### Mice

All mice were on a C57BL/6 background (table S1) (4, 22, 38, 69, 70) and bred in-house at the MRC ARES animal facility under specific pathogen-free conditions. Breeders were placed on breeding diet (ENVIGO, 2919), while experimental animals were on standard diet (SDS, 801722). All experimental mice used were females and euthanized between 11 to 12 weeks of age (unless otherwise indicated). All experiments were performed with littermates or independently verified with littermate controls. For survival studies, mice were monitored daily by experienced animal technicians blind to the genotypes, and culled when showing clinical signs of pale feet, piloerection, hunching and weight loss, which indicate a tumor burden resulting in intestinal obstruction and anemia as previously reported, and confirmed post-mortem. All animal experiments performed were under the approval of the UK Home Office.

### Generation of *Il25*-tomato gene-targeted mice

The *Il25*-knockout-tomato reporter mice were generated through recombineering. Neomycin-negative and Cre-recombinase-negative mice were backcrossed onto C57BL/6 background at least six times. Genotyping was through using PCR primers: (forward, GACAGAATTGCAGATGCTATTACTACGACC) and (reverse, ACCTCCTCGCCCTTGCTCACCAT) for the targeted product (380bp); (forward, GGCTAGGCTTCCAGGCTTCCAG) and (reverse, GGGGTTCTTGCTCTTTGCTGGG) for the wild-type product (200bp).

DNA fragments spanning from within the tdTomato cassette to beyond the ends of the arms of homology were amplified from genomic DNA from *Il25*^+/tom^ mice after the floxed neo cassette had been removed. Primers ASEQ8252 and ASEQ8253 were used to amplify the 5’ region and ASEQ8255 and ASEQ8256 for the 3’ region. These amplified fragments were then sequenced throughout their length to confirm the correct insertion of the tdTomato cassette at the IL25 start codon. Amplification and sequencing primers detailed in table S2.

### Recombinant cytokine treatment and IL-25R blockade in *Apc^1322T/+^* mice

For IL-25 treatment experiments, mice were injected intraperitoneally with 0.04 mg/kg of recombinant mouse IL-25 (Janssen) in PBS, three times per week for eight weeks, starting from three weeks of age. For IL-33 treatment experiments, mice were injected intraperitoneally with 0.03 mg/kg of recombinant mouse IL-33 (Biolegend) in PBS, three times per week for six weeks. For IL-25R (IL-17BR) blockade starting in young weaners, mice were injected intraperitoneally with 12.5 mg/kg of anti-IL-25R (clone D9.2) or control mIgG1 (LifeArc), twice a week for seven weeks, starting from three weeks of age. For IL-25R (IL-17BR) blockade starting in adults, mice were injected intraperitoneally with 12.5 mg/kg of anti-IL-25R (clone D9.2) or control mIgG1 (LifeArc), twice a week for four weeks, starting from seven weeks of age. After the final injection, mice were euthanized and samples collected for analysis.

### *Apc*-dextran sulfate sodium colonic tumor model and anti-IL-25R treatment

For induction of colonic adenocarcinoma in *Apc*^1322T/+^ mice, mice between five to six weeks of age were fed 1% DSS (MP Biomedicals, 160110) via drinking water for seven consecutive days, before reverting back to normal water. Mice were then monitored for five weeks, and euthanized and samples collected. For the therapeutic anti-IL-25R experiment for colonic tumors, one week after ending of the DSS treatment, mice were treated with 12.5 mg/kg of anti-IL-25R (clone D9.2) or control mIgG1 (LifeArc), twice a week for four weeks. After the final injection, mice were euthanized and samples collected for analysis.

### CD8-depletion, MDSC-depletion, and IFNγ neutralization

For depletion of CD8^+^ T cells *in vivo,* mice were injected with 12.5 mg/kg of anti-CD8α (Bio X Cell, BE0061) or control rIgG2b (Bio X Cell, BE0090) antibody, twice a week for eight weeks, starting from three weeks of age. For MDSC depletion, mice were treated with 12.5 mg/kg of anti-Gr1 (Bio X Cell, BE0075) or control rIgG2b (Bio X Cell, BE0090) antibody, three times a week for six weeks, starting from five weeks of age. For *in vivo* IFNγ neutralization, mice were injected with 12.5 mg/kg of anti-IFNγ (Bio X Cell, BE0055) or control rIgG1 (Bio X Cell, BE0088) antibody, twice a week for eight weeks, starting from three weeks of age. All injections were intraperitoneal. After the final injection, mice were euthanized and samples collected for analysis.

### Tumor scoring

Female (unless otherwise stated), background and age-matched mice were euthanized and tissues harvested. Small intestines and colons were flushed with cold PBS + 10 mM HEPES (Gibco), opened longitudinally, and fixed in methacarn (60% methanol, 30% chloroform and 10% acetic acid glacial) for 24 hours and subsequently washed with PBS. Tumor numbers were counted, and tumor size measured under a light microscope. The same protocol was adopted in the enumeration of colonic tumors in the *Apc*-DSS model. For tumor scoring, mouse genotypes and/or treatment conditions were blind to the examiner.

### Human samples

All tumor samples were surgically resected primary CRCs performed at Cambridge University Hospitals NHS Trust and processed on the same day for analysis. All patients gave written consent to participate in this study. The study was approved by the Wales Research Ethics Committee 7 (15/WA/0131).

### Cell isolation

Fresh tumors and 2 cm pieces of adjacent normal gut were collected separately from mice, followed by mechanical dissociation in RPMI-1640 containing 10mM HEPES (Gibco) and 2% foetal calf serum (FCS). Samples were digested with 62.5 μg/mL Liberase TL (Roche) and 0.125 KU/mL DNase I (Sigma) for 30 minutes at 37°C while shaking. Subsequently, cells were passed through a 50 μm filter into single cell suspension and washed with PBS + 2% FCS before antibody staining for flow cytometry. In experiments where the epithelial fraction and lamina propria were analyzed separately, the intestine was cut into 2 cm pieces and incubated with 1 mM dithiothreitol (Melford) and 5 mM EDTA in RPMI + 2% FCS + 10 mM HEPES for 2 x 20 minutes, shaking at 37°C, to collect the epithelial fraction. Subsequently, samples were digested with 62.5 μg/mL Liberase TL (Roche) and 0.125 KU/mL DNase I (Sigma) in RPMI + 2% FCS + 10 mM HEPES at 37°C, shaking, for 45 minutes to collect lamina propria cells. After digestion, cells were passed through a 70 μm cell strainer and washed with PBS + 2% FCS before antibody staining. Mesenteric lymph nodes were mechanically dissociated in RPMI, filtered through a 50 μm cell strainer, and washed with PBS + 2% FCS before antibody staining. Fresh, surgically-resected human CRC and adjacent normal tissue were collected in sterile PBS and transported on ice before sample processing. Samples were mechanically dissociated and digested with 100 μg/mL Liberase TM (Roche) and 0.2 KU/mL DNase I (Sigma) in RPMI, and incubated at 37°C for 30 minutes. Subsequently, cells were filtered through a 70 μm cell strainer and washed with PBS + 2% FCS before antibody staining for flow cytometry.

### Flow cytometry

For surface staining, single cell suspensions were blocked with anti-CD16/32 antibody (Fc block, clone 2.4G2) and Human TruStain FcX (Biolegend, 422302) for mouse and human samples, respectively, in PBS + 2% FCS, and incubated with fluorochrome-conjugated or biotinylated antibodies on ice for 30 minutes in the dark. Next, samples were incubated with fluorochrome-conjugated streptavidin on ice for a further 30 minutes if necessary. For intracellular and nuclear staining of mouse tumor samples, cells were fixed in 2% paraformaldehyde (ThermoFisher) for 45 minutes at room temperature in the dark, washed and subsequently stained with fluorochrome-conjugated antibodies for 45 minutes at room temperature in 1x Permeabilization Buffer (eBioscience, 00-8333-56). For IL-4 and IL-13 staining in ILC2s, and IFNγ, TNFα, perforin and granzyme staining in T cells, single cell suspensions were cultured with Cell Stimulation Cocktail (eBioscience, 00-4975-93) for 4 hours at 37°C, and subsequently stained with fluorochrome-conjugated antibodies as above for surface markers. After, cells were fixed using the BD Cytofix/Cytoperm Kit (BD Bioscience, 555028) and stained for intracellular cytokines according to the manufacture’s protocol. For intracellular and nuclear staining of human samples, cells were fixed and stained using the True-Nuclear Transcription Factor Buffer Set (Biolegend, 424401) according to the manufacturer’s protocol. Following intracellular staining, samples were washed serially with indicated 1 x Permeabilization Buffer and PBS + 2% FCS, and resuspended in PBS + 2% FCS for analyses through the BD LSRFortessa Flow Cytometer and FlowJo v10.2. All FACS sorting experiments in this manuscript were performed using an iCyt Synergy cell sorter (Sony Biotechnology), with >95% sort purity.

### Antibodies

For mouse experiments, antibodies used for surface staining include NK1.1 (PK136, BUV395), CD45 (30-F11, BV510), IL7Rα (SB/199, biotin), streptavidin BV605, CD8 (53-6.7, BV785 or FITC), CD11b (M1/70, FITC), KLRG1 (2F1, PerCP-eFluor 710), CD3 (145-2C11, PE-Cy7), CD11c (N418, PE-Cy7), CD19 (eBio1D3, PE-Cy7), Gr1 (RB6-8C5, PE-Cy7), FcεRI (MAR-1, PE-Cy7), TER119 (TER-119, PE-Cy7), CD4 (GK1.5, AF700), CD4 (RM4-5, BUV737), CD3 (17A2, eF450), CD11b (M1/70, eF450), CD11c (N418, eF450), CD19 (eBio1D3, eF450), F4/80 (BM8, eF450), Gr1 (RB6-8C5, eF450), FceRI (MAR-1, eF450), TCRβ (H57-597, eF450), TER119 (TER-119, eF450), ST2 (RMST2-2, AF488), streptavidin PE, CD11b (M1/70, BUV395), EpCAM (G8.8, BV421), Ly6C (HK1.4, BV711 or PE), Ly6G (1A8-Ly6g, PerCP-eFluor 710 or APC), MHC-II (M5/114.15.2, PE), TCRγδ (GL3, BV605), FceRI (MAR-1, FITC), CD11c (N418, PerCP-Cy5.5), CD317 (eBio927, APC), MHC-II (M5/114.15.2, BV510), CD19 (6D5, BV605), CD45 (30-F11, BV785), Gr1 (RB6-8C5, PerCP-Cy5.5), Siglec-F (E50-2440, PE), CD11c (N418, AF700), CD3 (17A2, BV510), CD8 (53-6.7, PE-Cy7), CD45 (30-F11, AF700), CD4 (GK1.5, PerCP-Cy5.5), TCRγδ (GL3, APC), IL17BR (D9.2, AF647), IL13RA1 (13MOKA, PE), IL4RA (mIL4R-M1, PE), ST2 (DJ8, FITC), CD11b (M1/70, BUV737), ICOS (7E.17G9, APC), NKp46 (29A1.4, eF660), streptavidin APC, Fixable Viability Dye eFluor 780 (eBioscience) and appropriate isotype controls. Lineage for mouse samples includes CD3, CD4, CD8, CD11b, CD11c, CD19, Gr1, FcεRI, NK1.1, and TER119, and in some cases individual lineage markers (eg. CD3, CD4, CD8, NK1.1, CD11b and Gr1 etc) were analyzed in a separate channel and not included in the main lineage cocktail as indicated in the figures. Antibodies used for intracellular and nuclear staining include GATA3 (L50-823, BV711), FOXP3 (FJK-16s, PE-Cy7), Tbet (eBio4B10, eF660), Ki67 (SolA15, PE-Cy7), IFNγ (XMG1.2, BV785), Granzyme (NGZB, PE or PE-Cy7), Perforin (S16009B, APC), Arginase 1 (A1exF5, PE), TNFα (MP6-XT22, AF700), IL-4 (11B11, PE), IL-13 (eBio13A, PE-Cy7), DCLK1 (Abcam, ab31704) and donkey-anti-rabbit AF647-conjugated antibody (Abcam, ab150075), and appropriate isotype controls.

For human samples, antibodies used for surface staining include IL17BR (D9.2, biotin), streptavidin BV421, CD56 (HCD56, BV650), CD4 (OKT4, BV785), CD45 (HI30, AF488), CRTH2 (BM16, PE), CD127 (A019D5, PE-Cy7), CD3 (OKT3, APC), FcεRI (AER-37(CRA-1), APC), CD11b (ICRF44, APC), CD11c (Bu15, APC), CD14 (HCD14, APC), CD19 (HIB19, APC), CD8 (RPA-T8, APC), CD123 (6H6, APC), CD14 (HCD14, Pacific Blue), CD86 (IT2.2, BV711), CD15 (W6D3, PerCP-Cy5.5), CD33 (WM53, PE), HLA-DR (L243, PE-Cy7), CD56 (HCD56, APC), CD45 (HI30, AF700), PD-L1 (29E.2A3, BV650), CD33 (WM53, BV785), CD155 (SKII.4, FITC), CD112 (TX31, PE), HVEM (122, PE-Cy7), CD8 (RPA-T8, BV510), CD3 (OKT3, Super Bright 645), PD1 (EH12.2H7, PerCP-Cy5.5), LAG3 (11C3C65, PE-Cy7), CD11b (ICRF44, FITC), Fixable Viability Dye eFluor 780 (eBioscience) and appropriate isotype controls. Lineage for human samples includes CD3, CD4, CD8, CD14, CD19, CD11b, CD11c, FcεRI, and CD123. Antibodies used for intracellular and nuclear staining include FOXP3 (206D, FITC), Tbet (eBio4B10, eF660), CTLA4 (L3D10, PE) and appropriate isotype controls.

### Fluorescence immunohistochemistry

Small intestine was dissected out and intestinal contents flushed with cold PBS. The small intestine was opened lengthways and cut into 3-cm long pieces, washed three times in cold PBS, and placed in 4% paraformaldehyde (ThermoFisher #28906) overnight at 4°C. Small intestine pieces were then washed with PBS and placed in 30% sucrose-PBS overnight. Swiss rolls were formed, embedded in 7.5% gelatin (Sigma) and 10% sucrose, and then flash frozen in a -40°C isopentane (Sigma) bath. The embedded Swiss rolls were then cryosectioned at 20 μm using a Leica CM3050 S Cryostat onto SuperFrost Plus Adhesion Slides (ThermoFisher #28906). Cryosections were washed three times with 1% Triton X-100 (BDH) in PBS. Sections were blocked with 2% goat serum (ab7481, Abcam) in PBS. Antibody staining was performed in blocking solution: anti-CD11b PE (BD Biosciences, 553311, M1/70), anti-Gr1 FITC (eBioscience, 11-5931-85, RB68C5), and anti-CD3 FITC (Biolegend, 100306, 145-2C11). Antibodies were added overnight at 4°C. Sections were washed three times for 15 minutes each with PBS. Slides were mounted in DAPI Fluoromount-G Mounting Medium (Southern Biotech).

### Microscopy and image processing

Immunofluorescence images were obtained using an LSM 710 inverted confocal microscope (Zeiss). Tumors were first identified using a 10x objective and imaged using 40x objective. Images were mounted using FIJI software, and annotations added using Adobe Illustrator.

### MDSC and T cell suppression assay

Intestinal ILC2s were sorted and cultured in complete RPMI (RPMI-1640 + 10% FCS + penicillin/streptomycin + 2-mercaptoethanol) with 10 ng/mL rm-IL-2 (Biolegend, 575406), 10 ng/mL rm-IL-7 (Biolegend, 577806), and 20 ng/mL rm-IL-25 (Janssen) for 3 days at 37°C, and supernatant collected (ILC2-SNT). ILC2-SNT were incubated with anti-IL-4 (Biolegend, 504122) and anti-IL-13 (eBioscience, 16-7135-85) neutralizing antibodies (αIL-4/13Ab-ILC2-SNT), or control rIgG1 (Biolegend, 400432 and eBioscience, 16-4301-85) antibodies (conAb-ILC2-SNT) for 1 hour on ice. Tumor M-MDSCs were sorted and incubated with αIL-4/13Ab-ILC2-SNT or conAb-ILC2-SNT for 2 hours at 37°C. Subsequently, sorted splenic CD8^+^ T cells (Live CD45^+^ TCRγδ^-^CD4^-^CD8^+^) stained with the CellTrace Violet Cell Proliferation Kit (ThermoFisher, C34557), were added to the ILC2-SNT-M-MDSC culture, and stimulated for 3 days with plate-coated anti-CD3ε (Biolegend, 100360) and 2 μg/mL soluble anti-CD28 (Bio X Cell, BE0015-1) at 37°C. Cell Stimulation Cocktail (eBioscience, 00-4975-93) was added for the last 4 hours of culture, before antibody staining. For Arginase 1 expression analysis in ILC2-SNT-treated M-MDSCs, tumor or splenic M-MDSCs were sorted and cultured with αIL-4/13Ab-ILC2-SNT or conAb-ILC2-SNT for 2 days, and stained with antibodies for flow cytometry.

### Adoptive transfer of ILC2s

Wild type or IL-13-deficient C57BL/6 mice were injected intraperitoneally with recombinant mouse IL-25 (1 μg/mouse) and IL-2/anti-IL-2 complex (IL-2, 0.5 μg/mouse, Biolegend; anti-IL-2, 0.25 μg/mouse, 2BScientific) for three consecutive days. Lymph nodes were collected and mechanically dissociated in RPMI, filtered through a 50 μm cell strainer, and washed with PBS + 2% FCS before antibody staining and FACS sorting for Lin^-^KLRG1^+^ICOS^+^ ILC2s. FACS purified ILC2s were cultured overnight in complete RPMI with 10 ng/mL rm-IL-7 and 20 ng/mL rm-IL-25, before injecting intravenously via the tail vein into DSS-treated ILC2-deficient *Apc*^1322T/+^ mice. Each injection consisted of 400,000 wild type or IL-13-deficient ILC2s in PBS as indicated. Each mouse received 4 injections, spaced 1 week apart, for the experiment comparing wild type ILC2 transfers to vehicle control and tissues collected one week after the final administration. All ILC2s for adoptive transfers were prepared fresh for each injection using the above protocol. For the experiment comparing wild type and IL-13-deficient ILC2 adoptive transfers, one injection with either wild type or IL-13-deficient ILC2s was performed and mice were analysed a week later.

### Single-cell RNA sequencing

Tumors were harvested and pooled from female mice between 77-83 days of age, and processed and FACS-sorted for the desired cell populations for library preparation. The 10x Genomics technology platform was used for 10x single-cell library preparation, and the 3’ libraries were obtained through the 10x Genomics Chromium Single Cell 3’ v3 protocol, and underwent subsequent sequencing. The reads were aligned to the mouse transcriptome (GRCm38), and expression was determined using the 10x Cell Ranger (version 3.0.2) wrapper for the STAR aligner (version 2.60a). Separate libraries were generated using FACS-sorted tumor cells (each consist of 2500 tumor CD45^-^EpCAM^+^ epithelial cells, 500 tumor CD45^+^CD11b^+^Ly6C^+^Ly6G^-^M-MDSCs, and 5000 CD45^+^ total immune cells) from ILC2-deficient (*Rora*^f/f^*Il7r*^Cre/+^) and Cre control (*Rora*^+/+^*Il7r*^Cre/+^) *Apc*^1322T/+^ mice, and then combined using Cell Ranger. Analysis and statistical calculations was performed using the 10x Genomics Loupe Browser (https://support.10xgenomics.com/single-cell-gene-expression/software/visualization/latest/what-is-loupe-cell-browser) and Enrichr (71–73).

### Human survival analysis

The publicly available TCGA Firehose Legacy Colorectal Adenocarcinoma dataset was analyzed using the online open-access cBioPortal resource (https://www.cbioportal.org/) (16, 17). All patient samples (222) with Agilent microarray mRNA data were included in the analysis, and the mRNA expression z-scores were calculated relative to all samples. CRC patients were separated into two groups based on tumor *IL25* expression above (*IL25*^high^) or below (*IL25*^low^) the cohort mean *IL25* expression. The two groups were compared for available survival data, including overall and disease-free survival. The same method was repeated for tumor *IL33* expression, and survival between *IL33*^high^ and *IL33*^low^ groups compared. Relapse-free survival and tumor *IL25* expression data by Marisa *et al*. (18) were analyzed using the online open-access R2: Genomics Analysis and Visualization Platform (http://r2.amc.nl). All patient samples (566) were included in the analysis, and segregated into two groups based on tumor *IL25* expression above (*IL25*^high^) and below (*IL25*^low^) the mean tumor *IL25* expression of all samples, and relapse-free survival compared. Graphs last analysed and retrieved from respective databases on 30^th^ March 2022.

### Statistical analysis

Statistical significance for human survival analyses was calculated automatically by the built-in system on cBioPortal and the R2 platform, for analysis of the TCGA Firehose Legacy (16, 17) and Marisa *et al.* 2013 (18) CRC datasets respectively. All other statistical analyses in this study were performed using the GraphPad Prism 9 software and detailed in the figure legends, unless indicated otherwise.

## Supplementary Material

Supplementary Material

## Data Availability

All data required to evaluate the conclusions of this study are present in the main text and the supplementary materials. Single-cell RNA sequencing data deposited in the Gene Expression Omnibus under GSE199113. Request for materials and correspondence should be addressed to Andrew McKenzie (anm@mrc-lmb.cam.ac.uk).

## One Sentence Summary

IL-25-activated ILC2s establish an immunosuppressive niche for CRC tumorigenesis through suppressing IFNγ-mediated anti-tumor immunity.
