# Supplementary material for: An innate IL-25-ILC2-MDSC axis creates a cancer-permissive microenvironment for Apc-mutation-driven intestinal tumorigenesis
Source: Sci Immunol. Author manuscript; Available in PMC 2022 Jun 7. (PMC7612821; doi:10.1126/sciimmunol.abn0175)
Supplement: Supplementary Material [file EMS144798-supplement-Supplementary_Material.pdf]

Supplementary Materials for

An innate IL-25-ILC2-MDSC axis creates a cancer-permissive microenvironment for *Apc*-mutation-driven intestinal tumorigenesis

Eric Jou[1,*], Noe Rodriguez-Rodriguez[1], Ana-Carolina F. Ferreira[1], Helen E. Jolin[1], Paula A. Clark[1], Kovilen Sawmynaden[2], Michelle Ko[1], Jane E. Murphy[1], Jonathan Mannion[1], Christopher Ward[3], David J. Matthews[2], Simon J. A. Buczacki[3] and Andrew N. J. McKenzie[1,4,*]

Correspondence to: anm@mrc-lmb.cam.ac.uk and ejou@mrc-lmb.cam.ac.uk

This PDF file includes:

Figs. S1 to S16
Supplementary Tables S1 and S2

Supplementary Figures

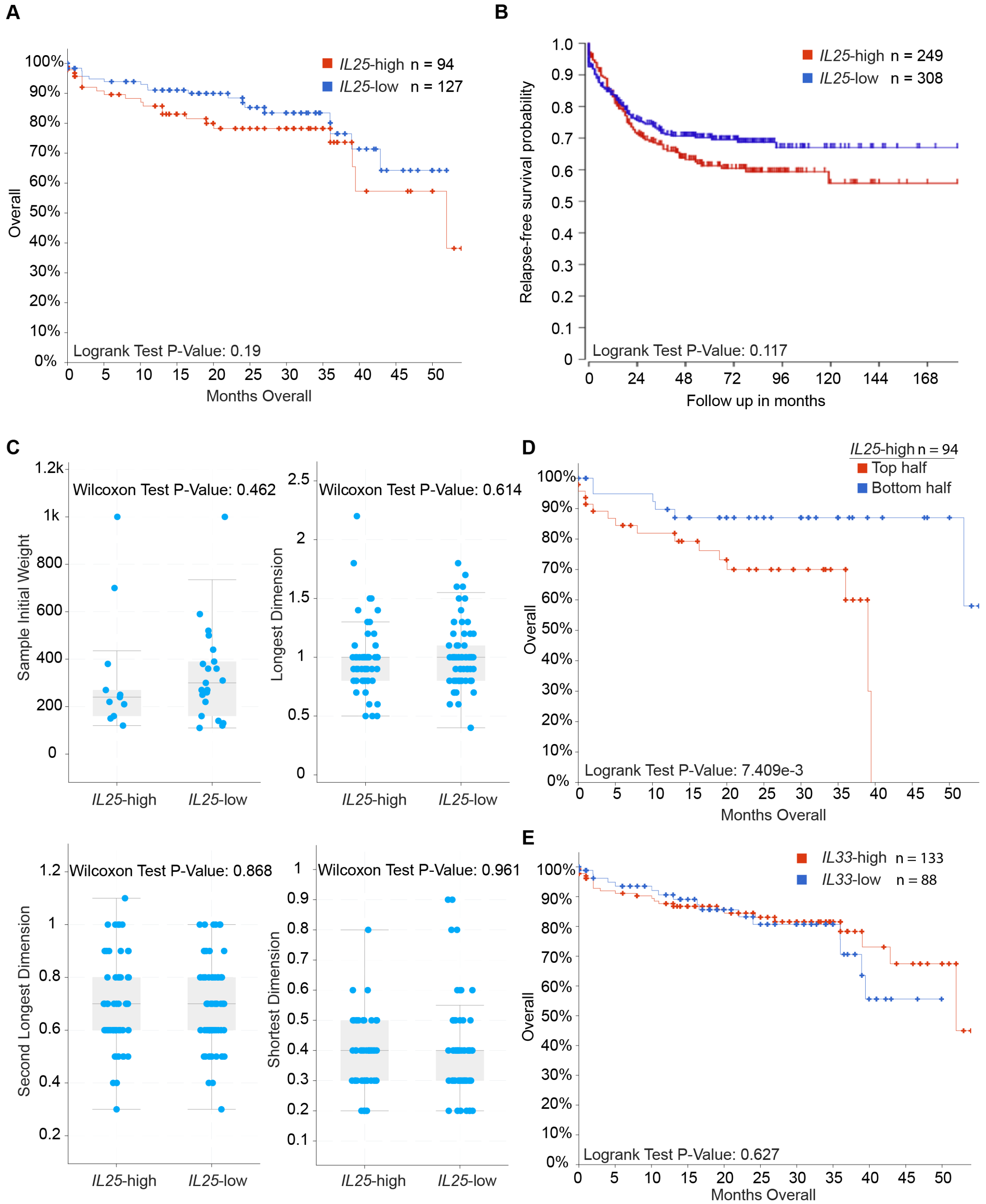

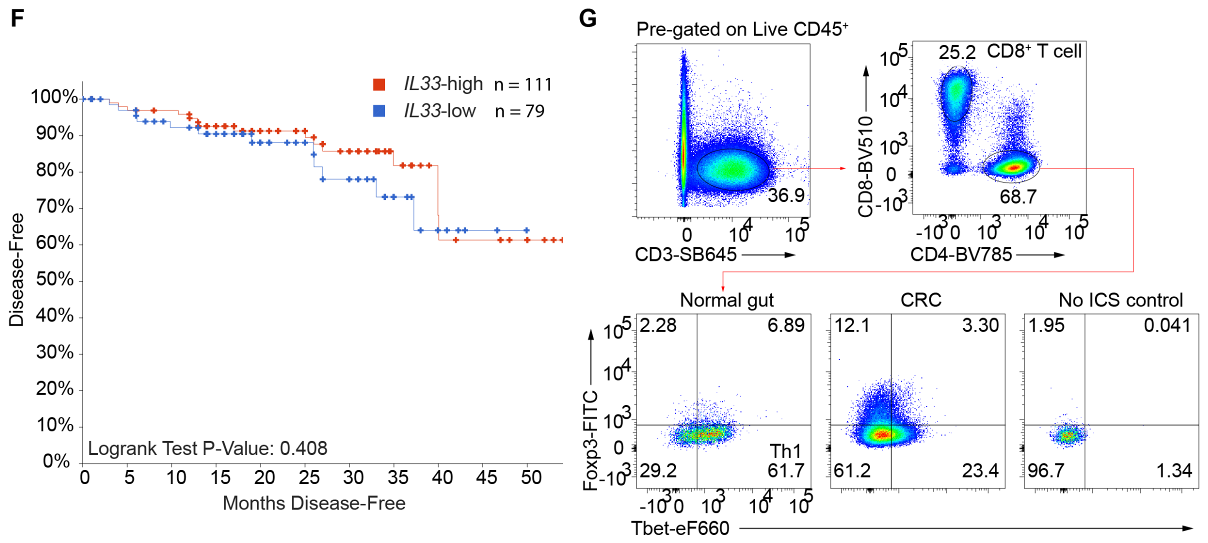

Fig. S1. High tumor *IL25* but not *IL33* expression is associated with poor survival in human CRC patients. (**A**) Overall survival of CRC patients from the TCGA Firehose Legacy microarray dataset, stratified into two groups by tumor *IL25* expression. *IL25*-high and *IL25*-low groups are defined as patients with CRC that had tumor *IL25* expression above and below the mean of the total samples, respectively. (**B**) Graph shows relapse-free survival of CRC patients from the large publicly available RNA dataset by Marisa *et al.* 2013 (*20*), analyzed through the R2 platform (http://r2.amc.nl), and stratified into two groups defined as above (*IL25*-high) or below (*IL25*-low) the overall cohort tumor *IL25* mean expression. (**C**) Weight and size measurements of CRC tumor samples from the TCGA Firehose Legacy microarray dataset, in the *IL25*-high and *IL25*-low groups defined as above and below the mean cohort tumor *IL25* expression respectively. (**D**) Overall survival of the *IL25*-high group (above cohort mean) from the TCGA dataset, further segregated into top and bottom half based on tumor *IL25* gene expression. (**E** and **F**) Overall (E) and disease-free (F) survival of CRC patients from the TCGA dataset, stratified into *IL33*-high or *IL33*-low group based on tumor *IL33* expression (*IL33*-high, above cohort mean; *IL33*-low, below cohort mean). (**G**) Gating strategy of CD8^{+} T cells and Th1 cells in human CRC and adjacent normal gut. SB645, Super Bright 645 fluorochrome. Statistical significance determined by log-rank test on cBioPortal (A, D, E and F) or R2 platform (B), and Wilcoxon test on cBioPortal (C).

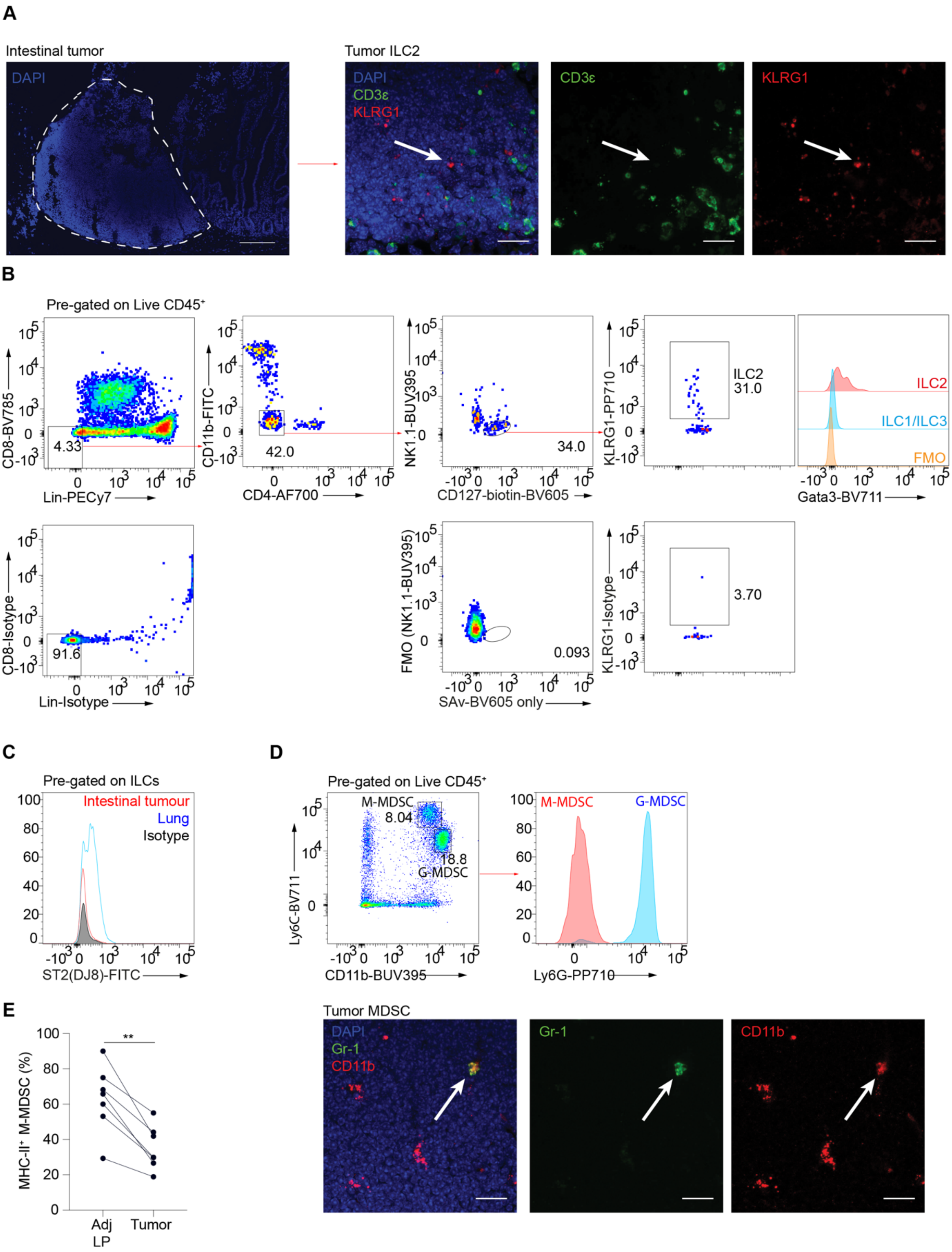
A
Intestinal tumor
DAPI
Tumor ILC2
DAPI
CD3ε
KLRG1
CD3ε
KLRG1
B
Pre-gated on Live CD45⁺
CD8-BV785
Lin-PECy7
4.33
CD11b-FITC
CD4-AF700
42.0
NK1.1-BUV395
CD127-biotin-BV605
34.0
KLRG1-PP710
ILC2
31.0
ILC2
ILC1/ILC3
FMO
Gata3-BV711
CD8-Isotype
Lin-Isotype
91.6
FMO (NK1.1-BUV395)
SAv-BV605 only
0.093
KLRG1-Isotype
3.70
C
Pre-gated on ILCs
Intestinal tumour
Lung
Isotype
ST2(DJ8)-FITC
D
Pre-gated on Live CD45⁺
M-MDSC
8.04
18.8
G-MDSC
Ly6C-BV711
CD11b-BUV395
M-MDSC
G-MDSC
Ly6G-PP710
E
MHC-II⁺ M-MDSC (%)
**
Adj LP
Tumor
Tumor MDSC
DAPI
Gr-1
CD11b
Gr-1
CD11b

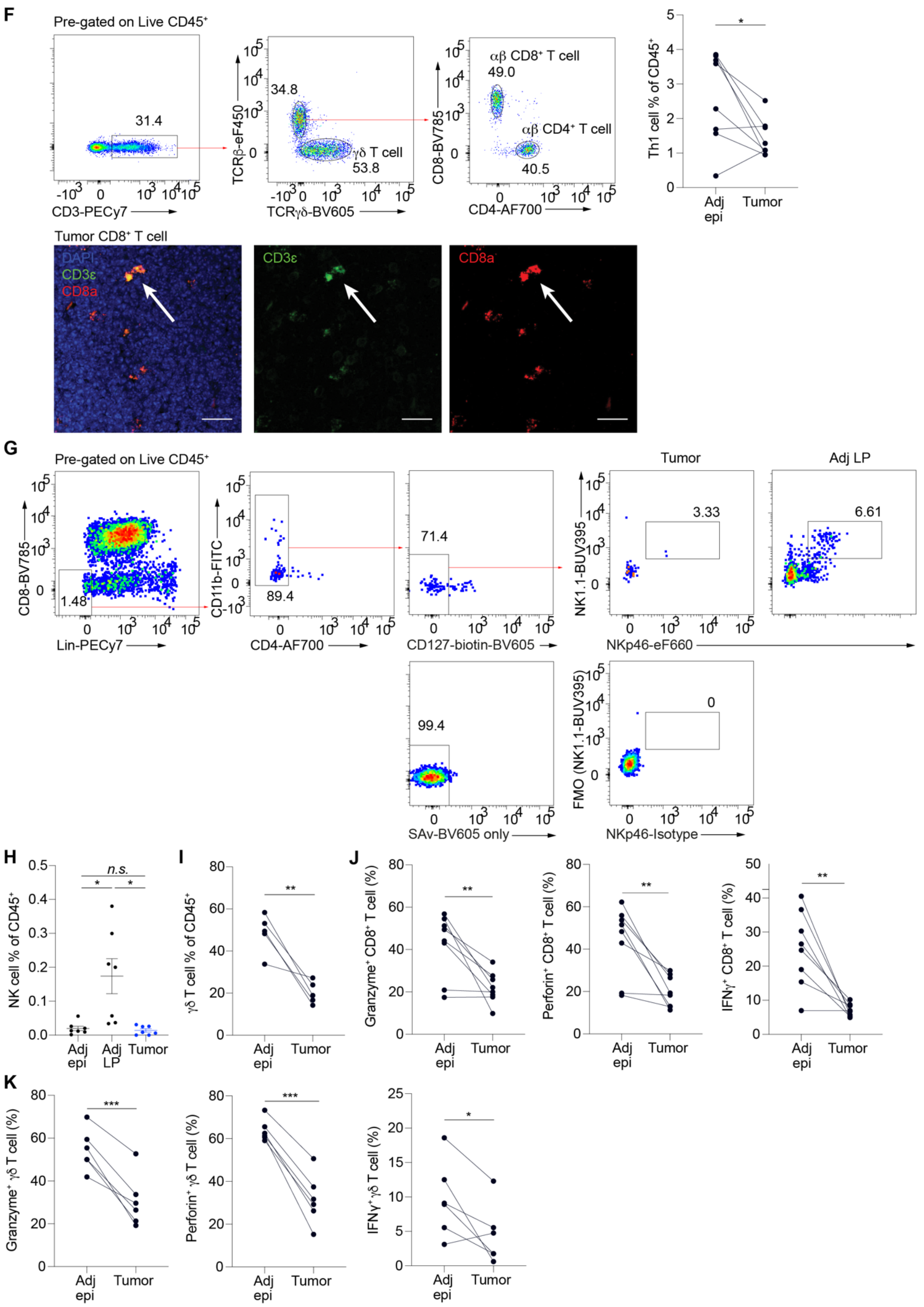

Fig. S2. Immunosuppressive tumor microenvironment in *Apc*$^{1322T/+}$ mouse intestinal tumors. (A) Representative images (left, 10x magnification, scale bar 150 μm) of intestinal tumor (dotted line) and

tumor infiltrating ILC2s (right, 40x magnification, scale bar 20 μm) from *Apc*$^{1322T/+}$ mice. (**B**) FACS plots show gating strategy of ILC2s in *Apc*$^{1322T/+}$ mice. (**C**) Plot shows ST2 (using antibody clone DJ8) expression on total ILCs in intestinal tumors and lung from *Apc*$^{1322T/+}$ mice. (**D**) FACS plots show gating strategy (top) and representative immunofluorescence images (bottom, 40x magnification, scale bar 20 μm) of tumor M-MDSCs and G-MDSCs. (**E**) Percentage MHC-II expression on adjacent lamina propria (Adj LP) and tumor M-MDSCs in *Apc*$^{1322T/+}$ mice (n = 7). (**F**) FACS plots show gating strategy of γδ, $CD4^+$ and $CD8^+$ T cells while graph shows Th1 cell frequency in tumor and adjacent epithelium (Adj epi) (n = 8), and immunofluorescence images show tumor infiltrating CD8 T cells (40x magnification, scale bar 20μm). (**G** and **H**) gating strategy (G) and frequency (H) of NK cells in indicated tissues from *Apc*$^{1322T/+}$ mice (n = 7). (**I**) Frequency of γδ T cells in and tumor in *Apc*$^{1322T/+}$ mice (n = 5). (**J**) Granzyme, perforin and IFNγ expression in $CD8^+$ T cells in tumor and Adj epi from *Apc*$^{1322T/+}$ mice (n = 8). (**K**) Granzyme, perforin and IFNγ expression in γδ T cells in tumor and Adj epi from *Apc*$^{1322T/+}$ mice (n = 6). Data pooled from two or more independent experiments and error bars show mean ± SEM. Statistical significance determined by paired two-tailed *t*-test, except in (H) by one-way ANOVA with Tukey's post hoc. *n.s.* non-signifiacnt, $^*P < 0.05$, $^{**}P < 0.01$, $^{***}P <$ 0.001.

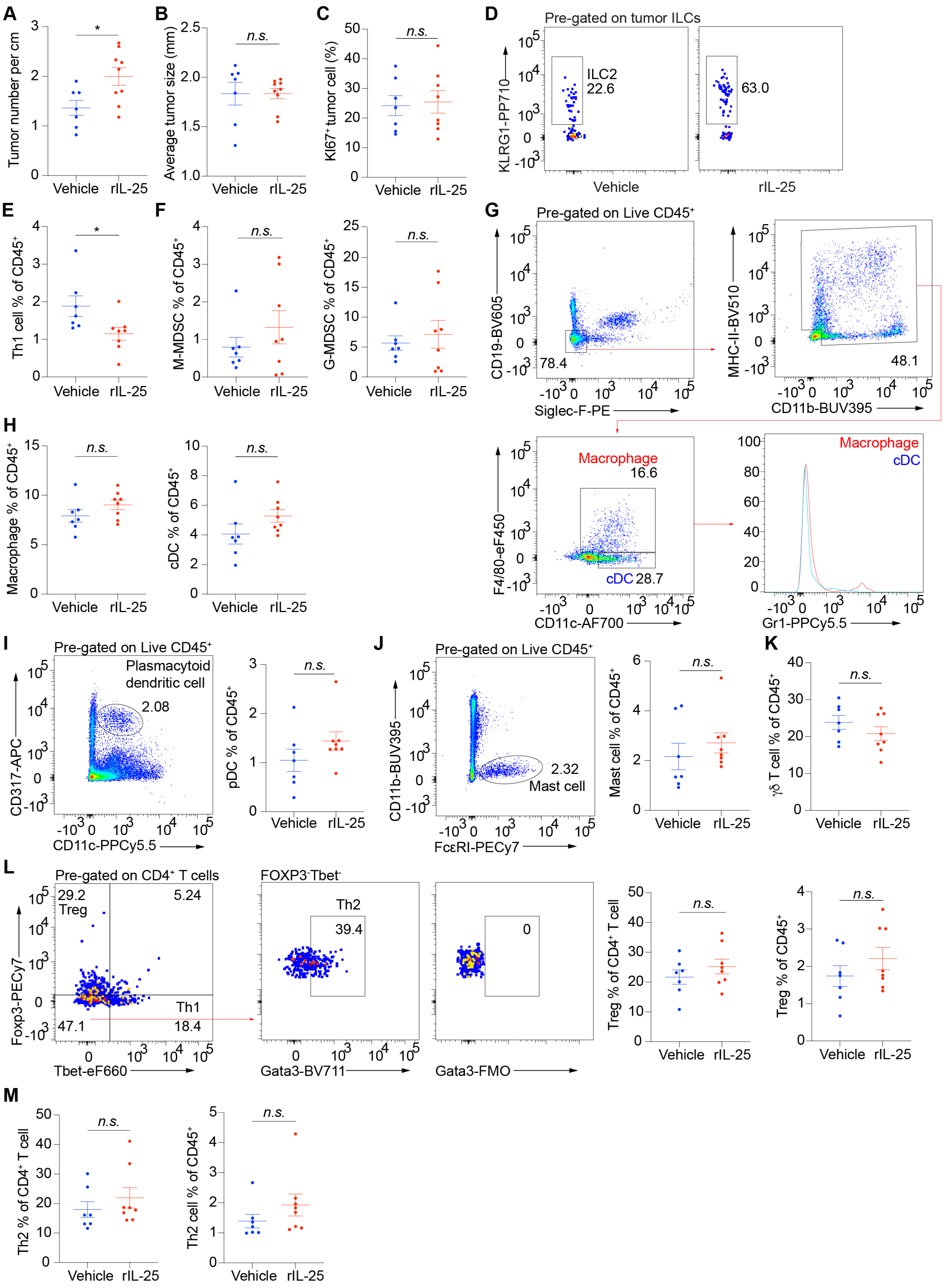

Fig. S3. IL-25 promotes tumors in *Apc*$^{1322T/+}$ mice. (**A** to **C**) Number of tumors per centimeter intestinal tissue (A) and average tumor size (B) (vehicle, n = 7; rIL-25, n = 9), and frequency of Ki67^{+}

EpCAM$^+$ tumor cells (C) (vehicle, *n* = 7; rIL-25, *n* = 8), in vehicle and rIL-25 treated *Apc*$^{1322T/+}$ mice. (**D**) Representative FACS plots showing tumor ILC2s in vehicle and rIL-25 treated *Apc*$^{1322T/+}$ mice. (**E** and **F**) Frequency of Th1 cells (E), M-MDSCs and G-MDSCs (F) in vehicle and rIL-25 treated *Apc*$^{1322T/+}$ mice (vehicle, *n* = 7; rIL-25, *n* = 8). (**G** to **J**) Gating strategy (G) and frequency of macrophages and conventional dendritic cells (cDC) (H), plasmacytoid dendritic cells (pDC) (I), and mast cells (J), in vehicle and rIL-25 treated *Apc*$^{1322T/+}$ mice (vehicle, *n* = 7; rIL-25, *n* = 8). (**K**) Frequency of tumor γδ T cells in vehicle and rIL-25 treated *Apc*$^{1322T/+}$ mice (vehicle, *n* = 7; rIL-25, *n* = 8). (**L**) Gating strategy of tumor Th1 cells, Th2 cells and Tregs, and frequency of Tregs, in vehicle and rIL-25 treated *Apc*$^{1322T/+}$ mice (vehicle, *n* = 7; rIL-25, *n* = 8). (**M**) Frequency of tumor Th2 cells in vehicle and rIL-25 treated *Apc*$^{1322T/+}$ mice (vehicle, *n* = 7; rIL-25, *n* = 8). Data pooled from two or more independent experiments and error bars show mean ± SEM. Statistical significance determined by unpaired two-tailed *t*-test. *n.s.* non-signifiacnt, $^*P < 0.05$.

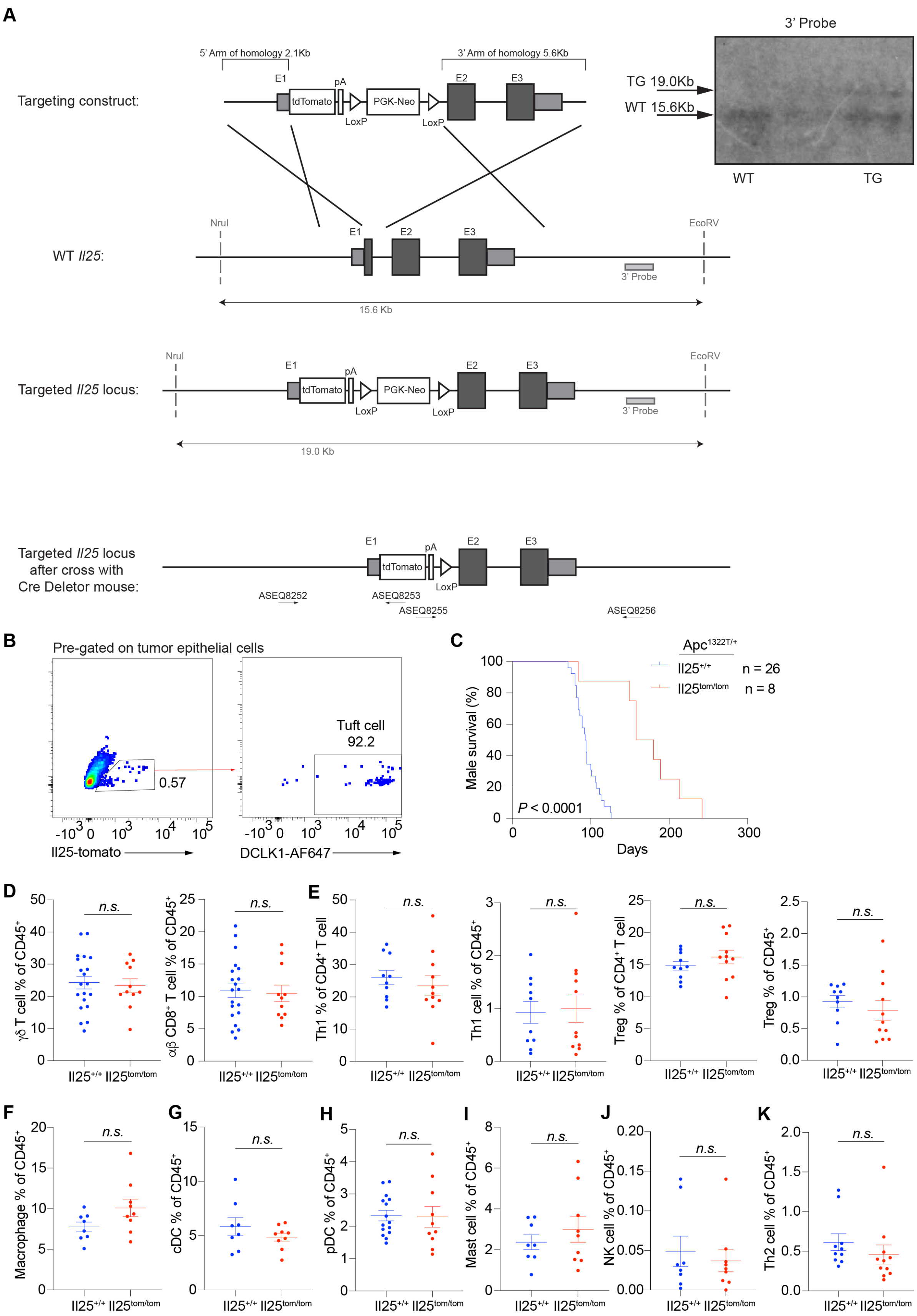

A
Targeting construct:
5' Arm of homology 2.1Kb
3' Arm of homology 5.6Kb
E1
tdTomato
pA
PGK-Neo
LoxP
E2
E3
3' Probe
TG 19.0Kb
WT 15.6Kb
WT
TG
WT Il25:
NruI
EcoRV
15.6 Kb
Targeted Il25 locus:
19.0 Kb
Targeted Il25 locus after cross with Cre Deletor mouse:
ASEQ8252
ASEQ8253
ASEQ8255
ASEQ8256
B
Pre-gated on tumor epithelial cells
0.57
Tuft cell 92.2
Il25-tomato
DCLK1-AF647
C
Apc1322T/+
Il25+/+ n = 26
Il25tom/tom n = 8
Male survival (%)
P < 0.0001
Days
D
γδ T cell % of CD45+
αβ CD8+ T cell % of CD45+
E
Th1 % of CD4+ T cell
Th1 cell % of CD45+
Treg % of CD4+ T cell
Treg % of CD45+
F
Macrophage % of CD45+
G
cDC % of CD45+
H
pDC % of CD45+
I
Mast cell % of CD45+
J
NK cell % of CD45+
K
Th2 cell % of CD45+
n.s.
Il25+/+ Il25tom/tom

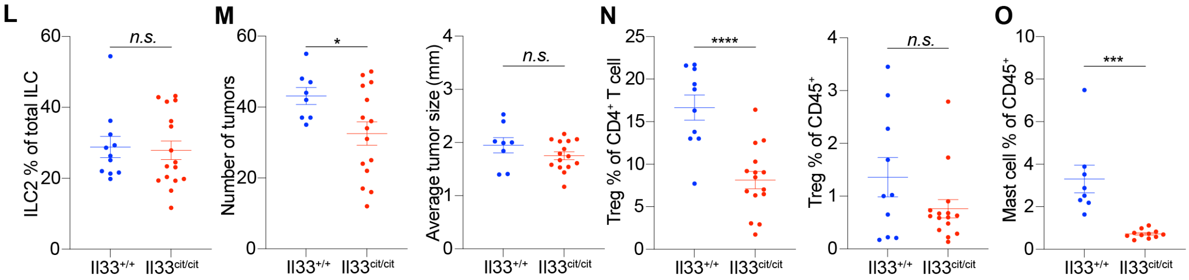

Fig. S4. IL-25-deficient *Apc*$^{1322T/+}$ mice show prolonged survival. (**A**) Schematic of *Il25*-tomato construct and Southern analysis. (**B**) Representative FACS plots showing DCLK1^{+} tuft cell frequency within *Il25*-tomato-expressing CD45^{-}EpCAM^{+} tumor epithelial cells in *Apc*$^{1322T/+}$ mice. (**C**) Survival of *Il25*$^{+/+}$ and *Il25*$^{tom/tom}$ *Apc*$^{1322T/+}$ male mice. (**D** to **K**) Frequency of tumor γδ and CD8^{+} T cells (*Il25*$^{+/+}$, *n* = 20; *Il25*$^{tom/tom}$, *n* = 11) (D), Th1 cells and Tregs (*Il25*$^{+/+}$, *n* = 10; *Il25*$^{tom/tom}$, *n* = 11) (E), macrophages (F) and conventional dendritic cells (cDC) (*Il25*$^{+/+}$, *n* = 8; *Il25*$^{tom/tom}$, *n* = 9) (G), plasmacytoid dendritic cells (pDC) (*Il25*$^{+/+}$, *n* = 15; *Il25*$^{tom/tom}$, *n* = 10) (H), mast cells (*Il25*$^{+/+}$, *n* = 8; *Il25*$^{tom/tom}$, *n* = 9) (I), NK cells (*Il25*$^{+/+}$, *n* = 8; *Il25*$^{tom/tom}$, *n* = 9) (J), and Th2 cells (*Il25*$^{+/+}$, *n* = 10; *Il25*$^{tom/tom}$, *n* = 11) (K) in *Il25*$^{+/+}$ and *Il25*$^{tom/tom}$ *Apc*$^{1322T/+}$ mice. (**L**) Frequency of tumor ILC2s in control (*Il33*$^{+/+}$) and IL-33-deficient (*Il33*$^{cit/cit}$) *Apc*$^{1322T/+}$ mice (*Il33*$^{+/+}$, *n* = 11; *Il33*$^{tom/tom}$, *n* = 16). (**M**) Number of tumors and average tumor size in control (*Il33*$^{+/+}$) and IL-33-deficient (*Il33*$^{cit/cit}$) *Apc*$^{1322T/+}$ mice (*Il33*$^{+/+}$, *n* = 8; *Il33*$^{tom/tom}$, *n* = 15). (**N** and **O**) Frequency of tumor Tregs (N) (*Il33*$^{+/+}$, *n* = 10; *Il33*$^{cit/cit}$, *n* = 15) and mast cells (O) (*Il33*$^{+/+}$, *n* = 8; *Il33*$^{cit/cit}$, *n* = 11) in control (*Il33*$^{+/+}$) and IL-33-deficient (*Il33*$^{cit/cit}$) *Apc*$^{1322T/+}$ mice. Data pooled from two or more independent experiments and error bars show mean ± SEM. FACS plots representative of more than 10 mice from at least two independent experiments. Statistical significance determined by two-sided log-rank test (C), and unpaired two-tailed *t*-test (D to O). *n.s.* non-signifiacnt, **P* < 0.05, ****P* < 0.001; *****P* < 0.0001.

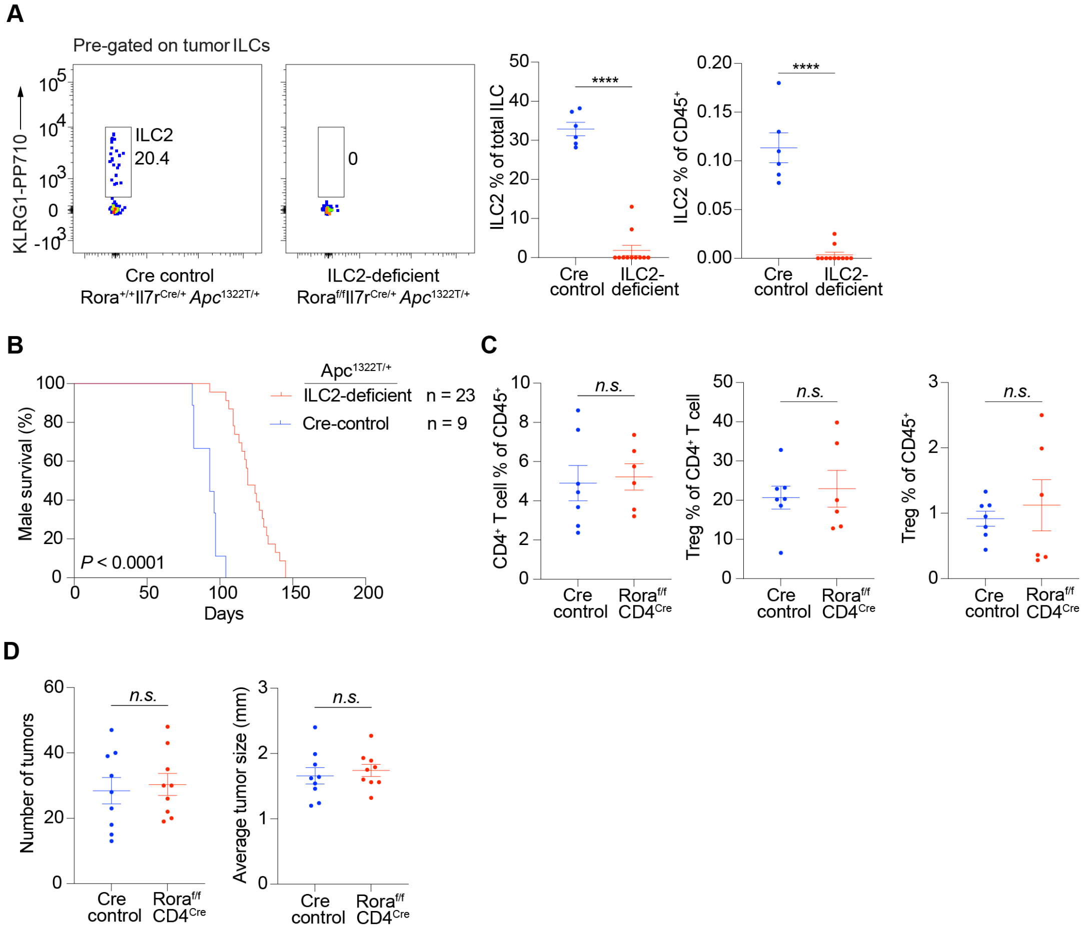

Fig. S5. Ablation of ILC2s protects from intestinal tumors and prolongs survival. (**A**) Representative FACS plots (left) and frequency (right) of ILC2s in ILC2-replete Cre-control (*Rora*$^{+/+}$*Il7r*$^{\text{Cre/+}}$ or *Rora*$^{+/\text{f}}$*Il7r*$^{\text{Cre/+}}$) and ILC2-deficient (*Rora*$^{\text{f/f}}$*Il7r*$^{\text{Cre/+}}$) *Apc*$^{\text{1322T/+}}$ mice (Cre-control, *n* = 6; ILC2-deficient, *n* = 11). (**B**) Survival of ILC2-deficient and Cre control *Apc*$^{\text{1322T/+}}$ male mice. (**C**) Frequency of tumor CD4$^+$ T cells and Tregs in *Rora*$^{\text{f/f}}$*Cd4*$^{\text{Cre/+}}$ (*n* = 6) and control *Rora*$^{+/\text{f}}$*Cd4*$^{\text{Cre/+}}$ (*n* = 7) *Apc*$^{\text{1322T/+}}$ mice. (**D**) Number of tumors and average tumor size of *Rora*$^{\text{f/f}}$*Cd4*$^{\text{Cre/+}}$ (*n* = 9) and control *Rora*$^{+/\text{f}}$*Cd4*$^{\text{Cre/+}}$ (*n* = 9) *Apc*$^{\text{1322T/+}}$ mice. Data representative of, or pooled from, two or more independent experiments and error bars show mean ± SEM. Statistical significance determined unpaired two-tailed *t*-test, except in (B) by two-sided log-rank test. *n.s.* non-signifiacnt, ****$P < 0.0001$.

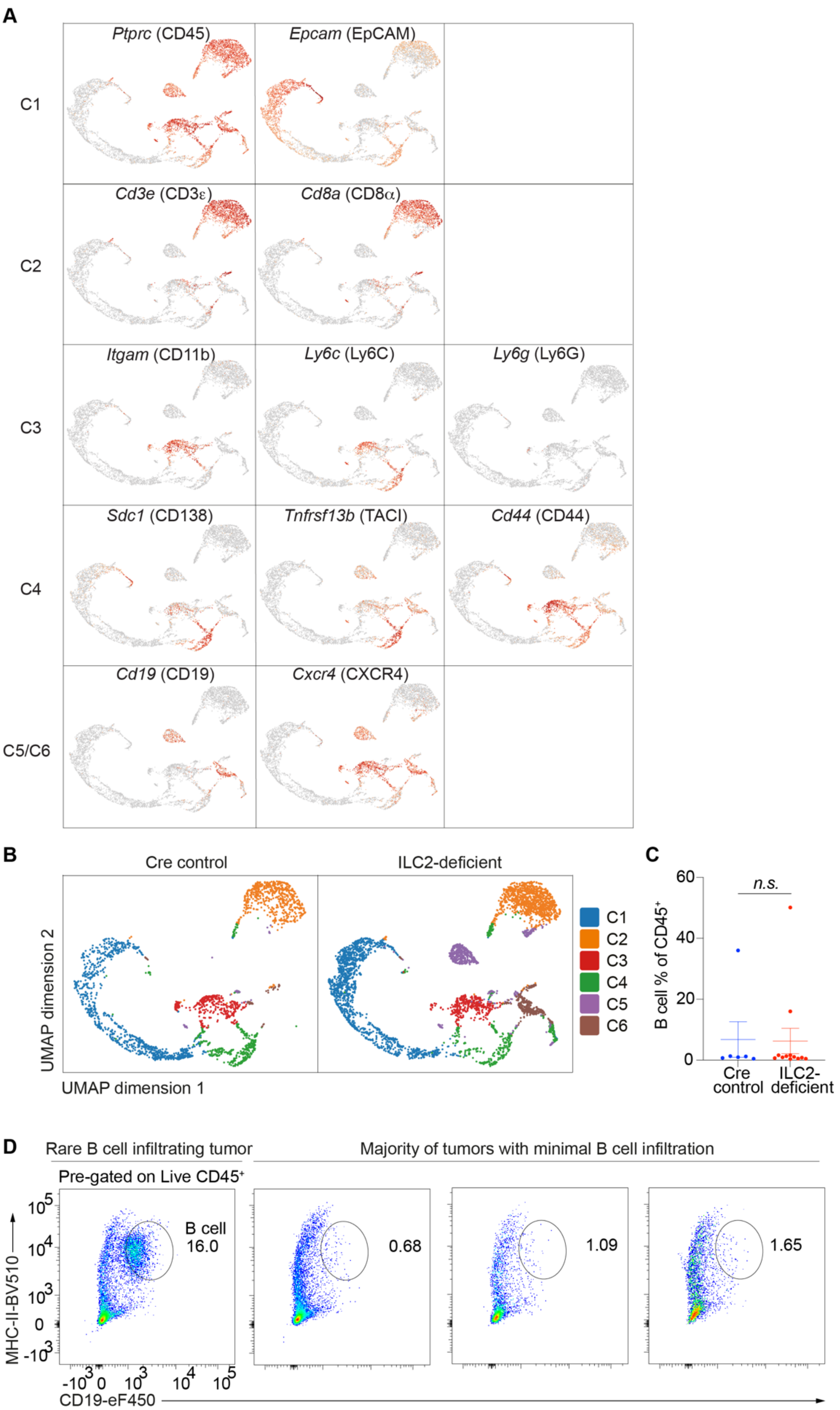

A
C1
C2
C3
C4
C5/C6
Ptprc (CD45)
Epcam (EpCAM)
Cd3e (CD3ε)
Cd8a (CD8α)
Itgam (CD11b)
Ly6c (Ly6C)
Ly6g (Ly6G)
Sdc1 (CD138)
Tnfrsf13b (TACI)
Cd44 (CD44)
Cd19 (CD19)
Cxcr4 (CXCR4)
B
Cre control
ILC2-deficient
UMAP dimension 2
UMAP dimension 1
C1
C2
C3
C4
C5
C6
C
B cell % of CD45+
60
40
20
0
n.s.
Cre control
ILC2-deficient
D
Rare B cell infiltrating tumor
Majority of tumors with minimal B cell infiltration
Pre-gated on Live CD45+
B cell
16.0
0.68
1.09
1.65
MHC-II-BV510
CD19-eF450

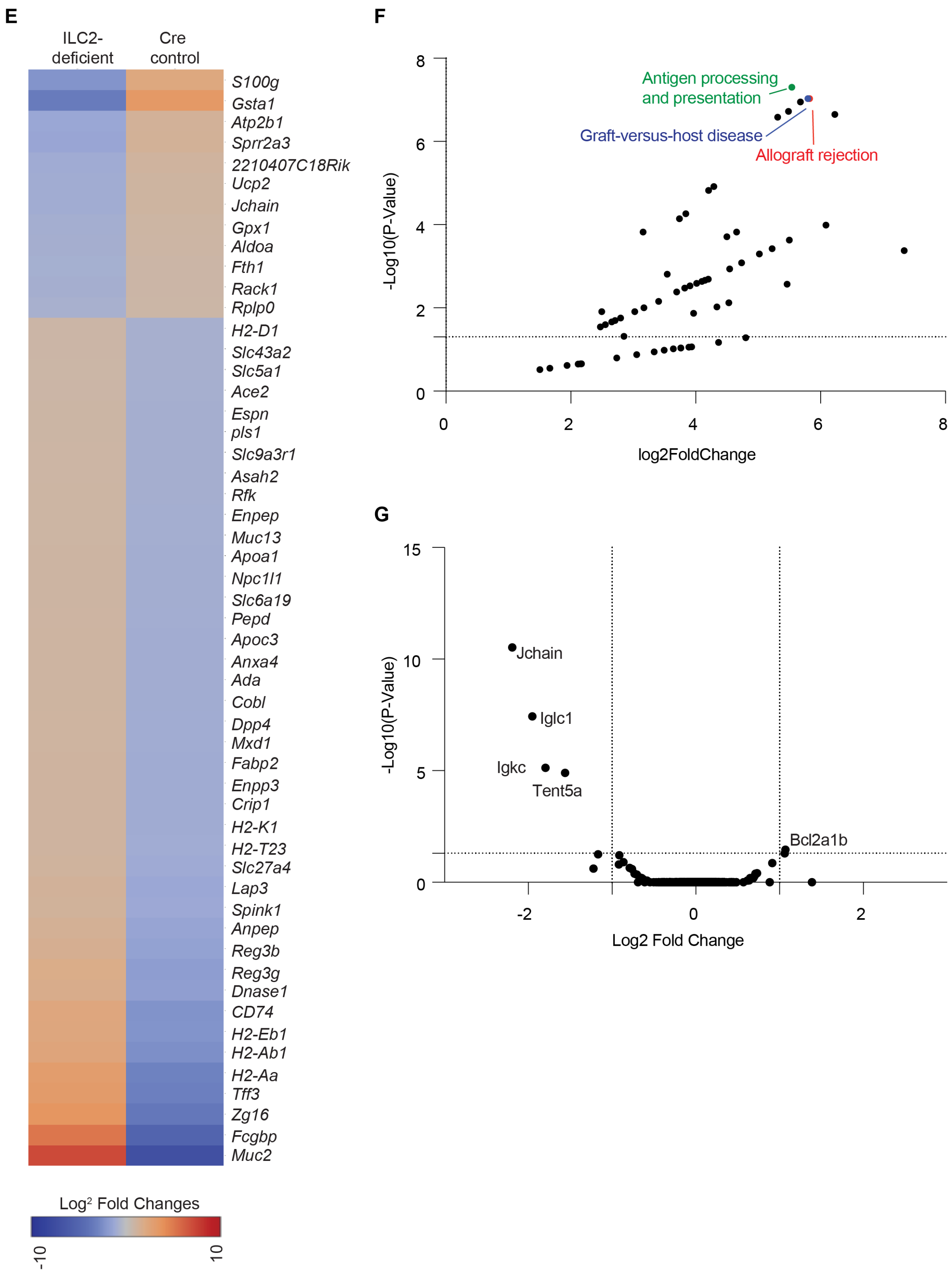

Fig. S6. ILC2 deficiency does not affect tumor B cell infiltration. (**A**) UMAP plot of single-cell RNAseq (scRNAseq) analysis showing expression of indicated genes (encoded protein shown in brackets). (**B**) UMAP plot of scRNAseq analysis of tumor epithelial cells (C1; $CD45^{-}EpCAM^{+}$), M-MDSCs (C3; $CD45^{+}CD11b^{+}Ly6C^{+}Ly6G^{-}$) and total immune population (remaining clusters; $CD45^{+}$)

sorted from intestinal tumors from ILC2-deficient and Cre control *Apc*$^{1322T/+}$ mice. (**C**) Frequency of tumor B cells in Cre control and ILC2-deficient *Apc*$^{1322T/+}$ mice from flow cytometry analysis (Cre control, $n = 6$; ILC2-deficient, $n = 12$). (**D**) Representative FACS plots showing tumor B cells in individual *Apc*$^{1322T/+}$ mouse tumors. (**E** and **F**) Heatmap (E) and KEGG pathway analysis (F) of differentially expressed genes identified through scRNAseq, between tumor epithelial cells from ILC2-deficient and Cre control *Apc*$^{1322T/+}$ mice. (**G**) Plot shows differentially expressed genes identified through scRNAseq, by tumor $CD8^+$ T cells (C2) from ILC2-deficient *Apc*$^{1322T/+}$ mice relative to Cre-control *Apc*$^{1322T/+}$ mice. Data representative of, or pooled from, two or more independent experiments and error bars show mean ± SEM (C). Statistical significance determined by unpaired two-tailed *t*-test (C), and (E and F) using the10x Genomics Loupe Browser and Enrichr. *n.s.* non-signifiacnt.

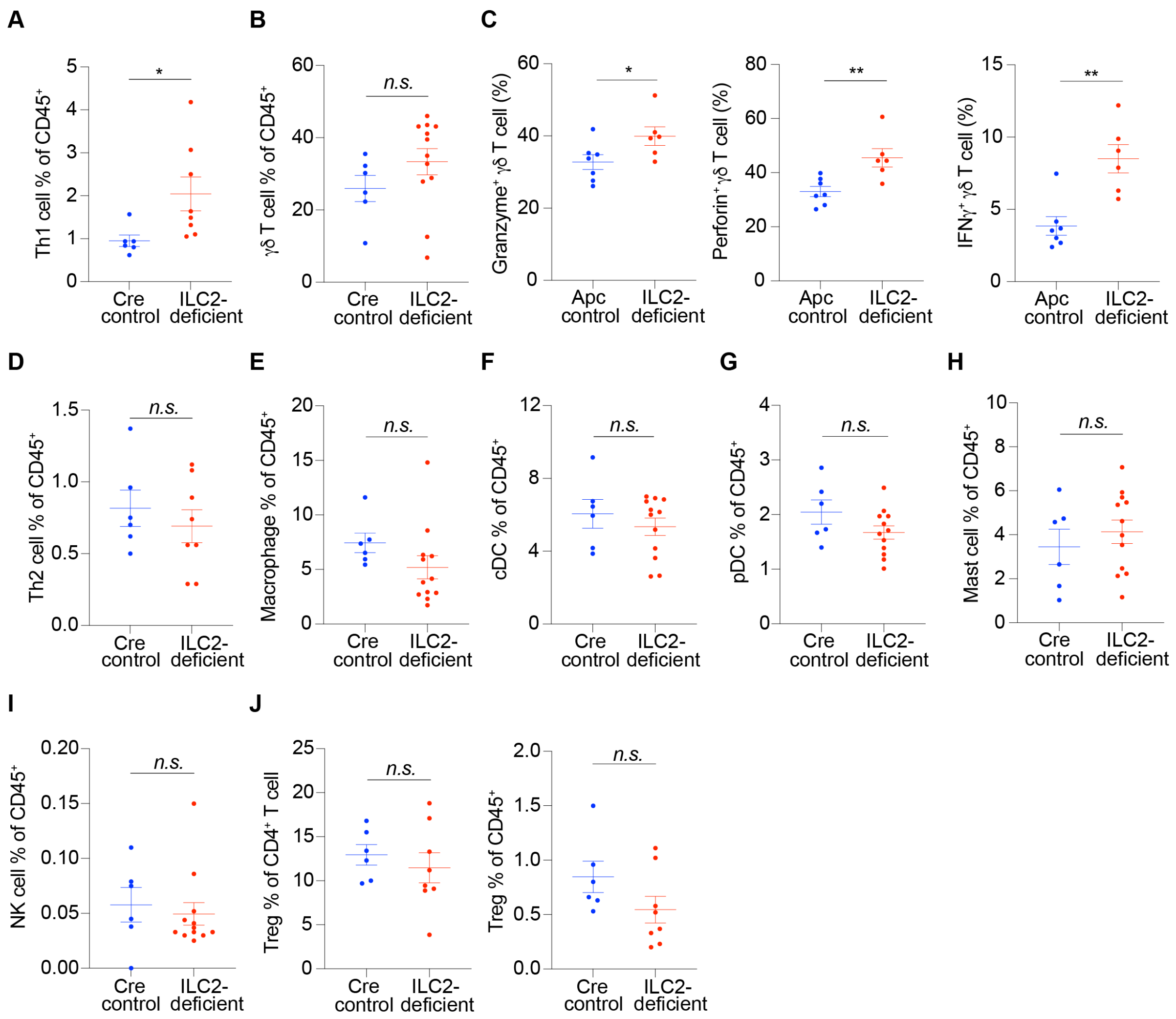

Fig. S7. Ablation of ILC2s improves anti-tumor immunity. (**A**) Frequency of tumor Th1 cells (Cre control, *n* = 6; ILC2-deficient, *n* = 8) in ILC2-deficient and Cre control *Apc*$^{1322T/+}$ mice. (**B** and **C**) Frequency (Cre control, *n* = 6; ILC2-deficient, *n* = 12) (A), and granzyme, perforin and IFNγ expression (*Rora*$^{+/+}$*Il7r*$^{+/+}$ control, *n* = 7; ILC2-deficient, *n* = 6) (B), in tumor γδ T cells from ILC2-deficient and control *Apc*$^{1322T/+}$ mice. (**D**) Frequency of tumor Th2 cells (Cre control, *n* = 6; ILC2-deficient, *n* = 8) in ILC2-deficient and Cre control *Apc*$^{1322T/+}$ mice. (**E** to **I**) Frequency of macrophages (E), conventional dendritic cells (cDC) (F), plasmacytoid dendritic cells (pDC) (G), mast cells (H), and NK cells (I), in ILC2-deficient and control *Apc*$^{1322T/+}$ mice (Cre control, *n* = 6; ILC2-deficient, *n* = 12). (**J**) Frequency of tumor Tregs in ILC2-deficient and control *Apc*$^{1322T/+}$ mice (Cre control, *n* = 6; ILC2-deficient, *n* = 8). Data pooled from two or more independent experiments and error bars show mean ± SEM. Statistical significance determined by unpaired two-tailed t-test. *n.s.* non-signifiacnt, **P* < 0.05, ***P* < 0.01.

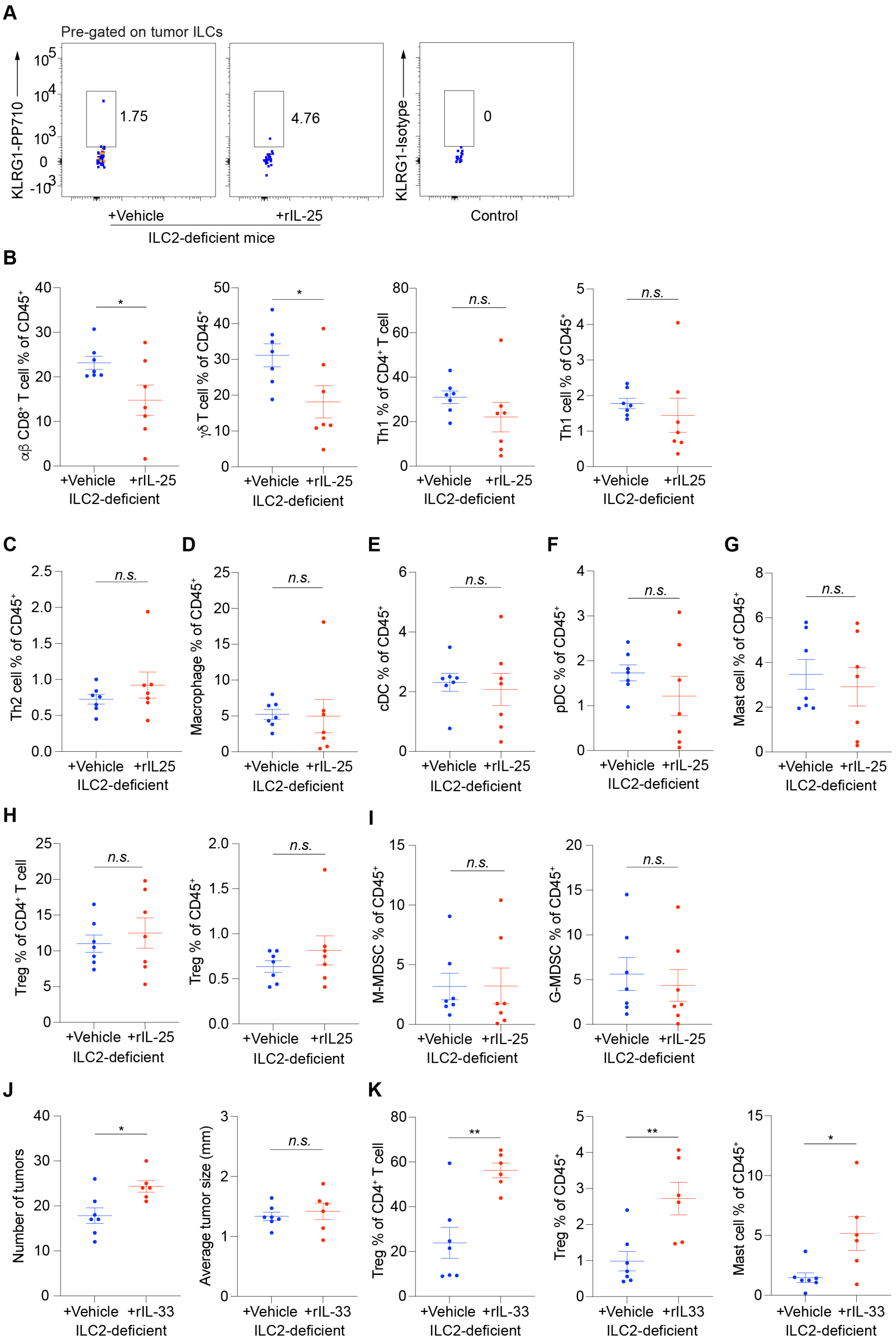

Fig. S8. IL-33 treatment in ILC2-deficient mice increases intestinal tumorigenesis. (**A**) Representative FACS plots showing (lack-of) ILC2s in vehicle and rIL-25 treated ILC2-deficient *Apc*$^{1322T/+}$ mice. (**B** to **I**) Frequency of tumor CD8^{+}, γδ and Th1 T cells (B), Th2 cells (C),

macrophages (D), conventional dendritic cells (cDC) (E), plasmacytoid dendritic cells (pDC) (F), mast cells (G), Tregs (H), and M-MDSCs and G-MDSCs (I), in vehicle and rIL-25 treated ILC2-deficient *Apc*$^{1322T/+}$ mice (vehicle, *n* = 7; rIL-25, *n* = 7). (**J**) Number of tumors and average tumor size in vehicle and rIL-33 treated ILC2-deficient *Apc*$^{1322T/+}$ mice (vehicle, *n* = 7; rIL-25, *n* = 6). (**K**) Frequency of tumor Tregs and mast cells in vehicle and rIL-33 treated ILC2-deficient *Apc*$^{1322T/+}$ mice (vehicle, *n* = 7; rIL-25, *n* = 6). Data pooled from two or more independent experiments and error bars show mean ± SEM. Statistical significance determined by unpaired two-tailed *t*-test. *n.s.* non-signifiacnt, *$P < 0.05$, **$P < 0.01$.

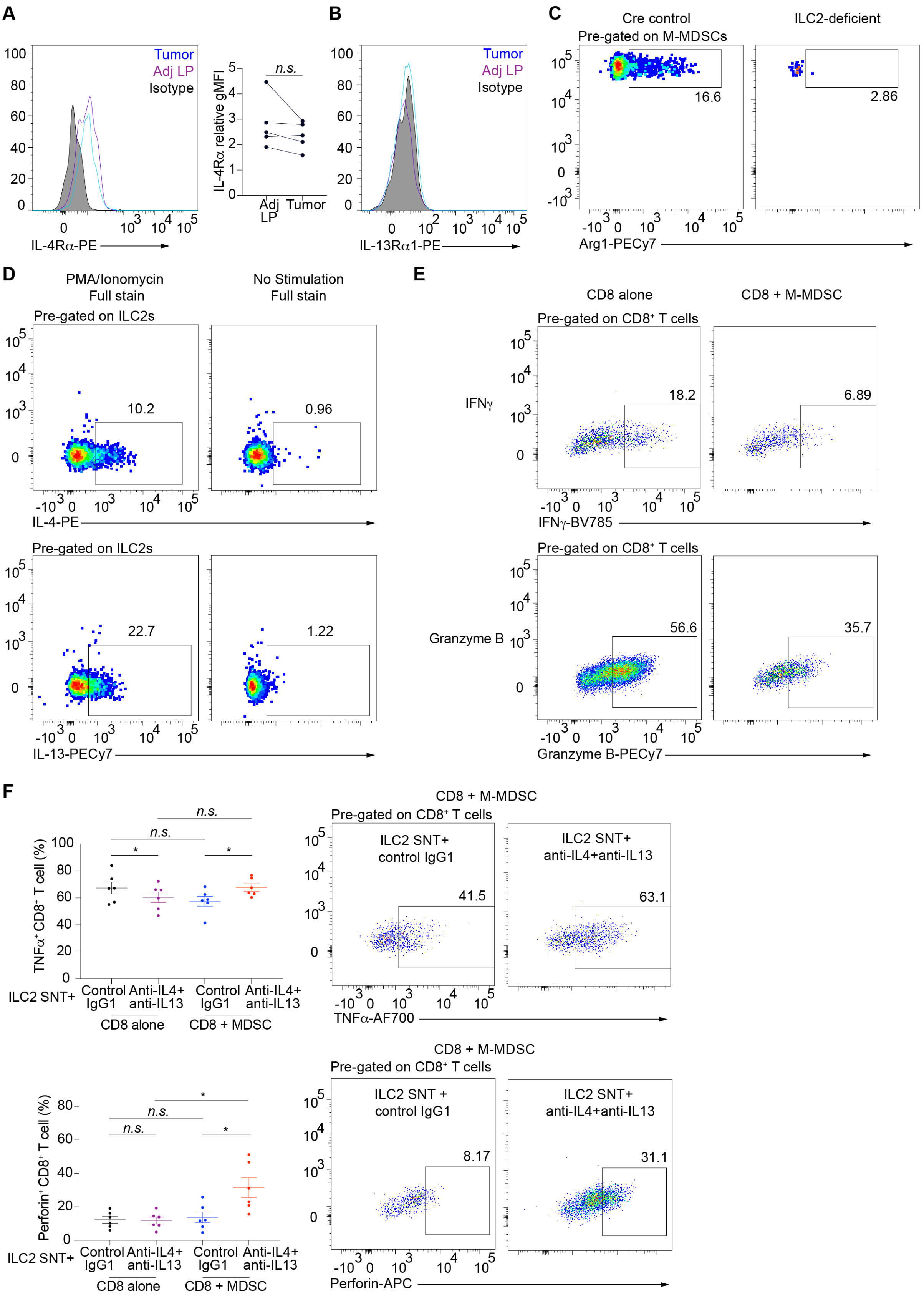

A
Tumor
Adj LP
Isotype
IL-4Rα-PE
IL-4Rα relative gMFI
n.s.
Adj LP
Tumor
B
Tumor
Adj LP
Isotype
IL-13Rα1-PE
C
Cre control
ILC2-deficient
Pre-gated on M-MDSCs
16.6
2.86
Arg1-PECy7
D
PMA/Ionomycin
Full stain
No Stimulation
Full stain
Pre-gated on ILC2s
10.2
0.96
IL-4-PE
Pre-gated on ILC2s
22.7
1.22
IL-13-PECy7
E
CD8 alone
CD8 + M-MDSC
Pre-gated on CD8+ T cells
IFNγ
18.2
6.89
IFNγ-BV785
Pre-gated on CD8+ T cells
Granzyme B
56.6
35.7
Granzyme B-PECy7
F
TNFα+ CD8+ T cell (%)
n.s.
n.s.
*
*
ILC2 SNT+
Control IgG1
Anti-IL4+ anti-IL13
Control IgG1
Anti-IL4+ anti-IL13
CD8 alone
CD8 + MDSC
CD8 + M-MDSC
Pre-gated on CD8+ T cells
ILC2 SNT+ control IgG1
ILC2 SNT+ anti-IL4+anti-IL13
41.5
63.1
TNFα-AF700
Perforin+ CD8+ T cell (%)
*
n.s.
n.s.
*
ILC2 SNT+
Control IgG1
Anti-IL4+ anti-IL13
Control IgG1
Anti-IL4+ anti-IL13
CD8 alone
CD8 + MDSC
CD8 + M-MDSC
Pre-gated on CD8+ T cells
ILC2 SNT + control IgG1
ILC2 SNT+ anti-IL4+anti-IL13
8.17
31.1
Perforin-APC

G

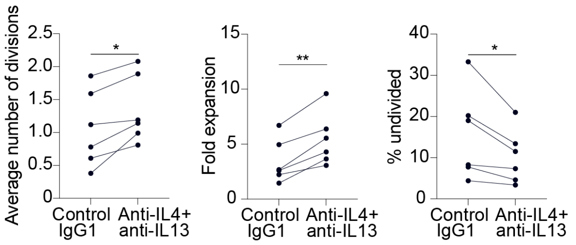

Fig. S9. ILC2-derived IL-4 and IL-13 promotes M-MDSC-mediated CD8[+] T cell suppression. (**A**) Representative FACS plot and quantification (relative gMFI) of IL-4Rα receptor expression on G-MDSCs from *Apc*$^{1322T/+}$ mice (n = 5). Relative gMFI, geometric mean fluorescent intensity relative to isotype control. (**B**) Representative FACS plot showing IL-13Rα1 receptor staining on G-MDSCs from *Apc*$^{1322T/+}$ mice. (**C**) Representative FACS plots showing Arginase 1 (Arg1) expression in tumor M-MDSCs from ILC2-deficient and Cre control *Apc*$^{1322T/+}$ mice. (**D**) Representative FACS plots showing IL-4 and IL-13 expression in rIL-25-stimulated cultured intestinal ILC2s. (**E**) Representative FACS plots showing IFNγ (top) and granzyme B (bottom) expression in CD8^{+} T cells cultured alone or with tumor M-MDSCs, in the presence of conAb-ILC2-SNT. (**F**) TNFα and perforin expression in CD8^{+} T cells cultured alone or with tumor M-MDSCs, in the presence of αIL-4/13Ab-ILC2-SNT or conAb-ILC2-SNT (n = 6). (**G**) Quantification of CD8^{+} T cell proliferation measured by average number of divisions, fold expansion, and percentage undivided, when cocultured with tumor M-MDSCs treated with αIL-4/13Ab-ILC2-SNT or conAb-ILC2-SNT (n = 6). αIL-4/13Ab-ILC2-SNT and conAb-ILC2-SNT, ILC2-supernatent (ILC2-SNT) pre-treated with anti-IL-4 and anti-IL-13 neutralizing antibodies, or control antibody, respectively. Data representative of or pooled from two or more independent experiments. Statistical significance determined by paired two-tailed *t*-test (A and G), and one-way ANOVA with Tukey's post hoc (F). *n.s.* non-signifiacnt, $*P < 0.05$, $**P < 0.01$.

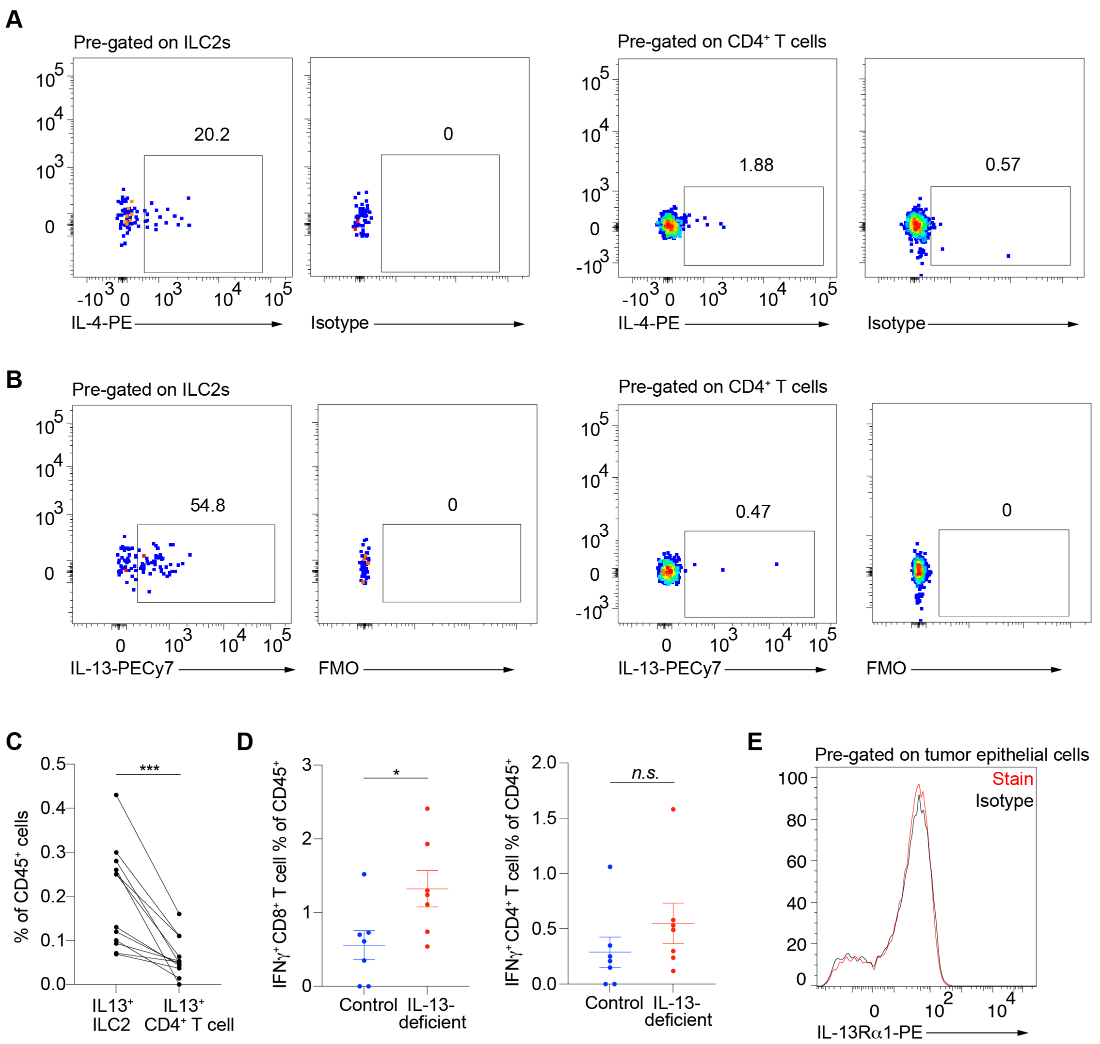

Fig. S10. Tumor ILC2s are major producers of IL-4 and IL-13. (**A**) Representative FACS plots showing IL-4 expression by tumor ILC2s (left) and CD4$^+$ T cells (right) from *Apc*$^{1322T/+}$ mice. (**B**) Representative FACS plots showing IL-13 expression by tumor ILC2s (left) and CD4$^+$ T cells (right) from *Apc*$^{1322T/+}$ mice. (**C**) Frequency of IL-13$^+$ tumor ILC2s and CD4$^+$ T cells as percentage of CD45$^+$ cells from *Apc*$^{1322T/+}$ mice (n = 12). (**D**) Tumor IFNγ$^+$ CD8$^+$ and CD4$^+$ T cell frequency in control and IL-13-deficient *Apc*$^{1322T/+}$ mice (control, n = 7; IL-13-deficient, n = 7). (**E**) Representative FACS plot showing IL-13Rα1 receptor staining on tumor epithelial cells from *Apc*$^{1322T/+}$ mice. FACS plots representative of more than 10 mice from at least two independent experiments. *n.s.* non-signifiacnt, *$P < 0.05$, ***$P < 0.001$.

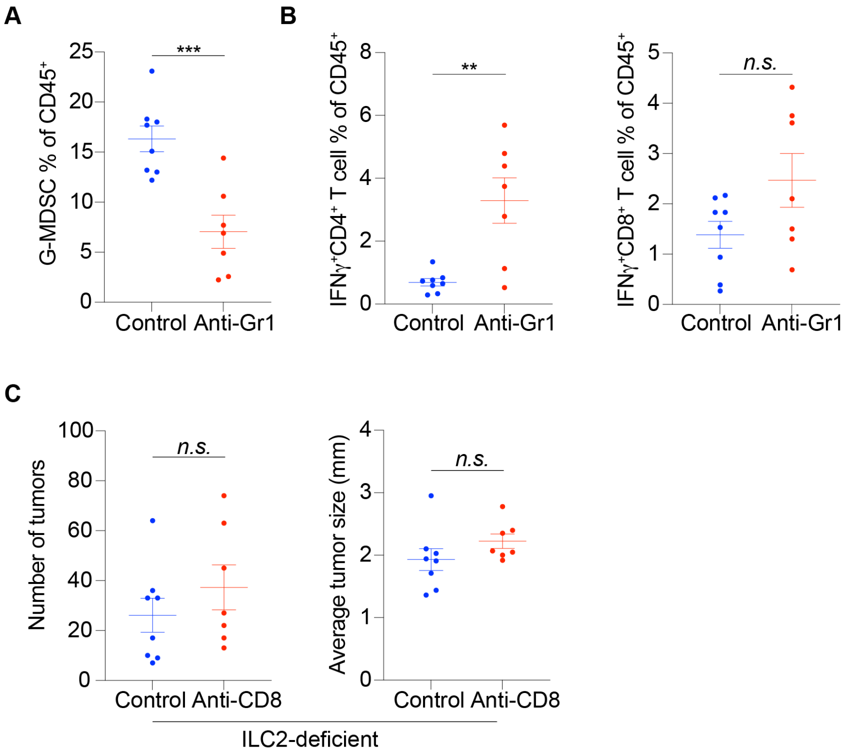

Fig. S11. Anti-GR-1 treatment depletes MDSCs and enhances tumor infiltrating T cell IFNγ expression in *Apc*$^{1322T/+}$ mice. (**A**) Frequency of G-MDSCs in control rIgG2b and anti-Gr1 treated *Apc*$^{1322T/+}$ mice (control, n = 8; treatment, n = 7). (**B**) Tumor IFNγ^{+} CD4^{+} and CD8^{+} T cell frequency in control and anti-Gr1 treated *Apc*$^{1322T/+}$ mice (control, n = 8; treatment, n = 7). (**C**) Number of tumors and average tumor size in control rIgG2b or anti-CD8α treated ILC2-deficient (*Rora*$^{f/f}$*Il7r*$^{Cre/+}$) *Apc*$^{1322T/+}$ mice (control, n = 8; anti-IFNγ, n = 7). Data pooled from two or more independent experiments and error bars show mean ± SEM. Statistical significance determined by unpaired two-tailed *t*-test. *n.s.* non-signifiacnt, $^{**}P < 0.01$, $^{***}P < 0.001$.

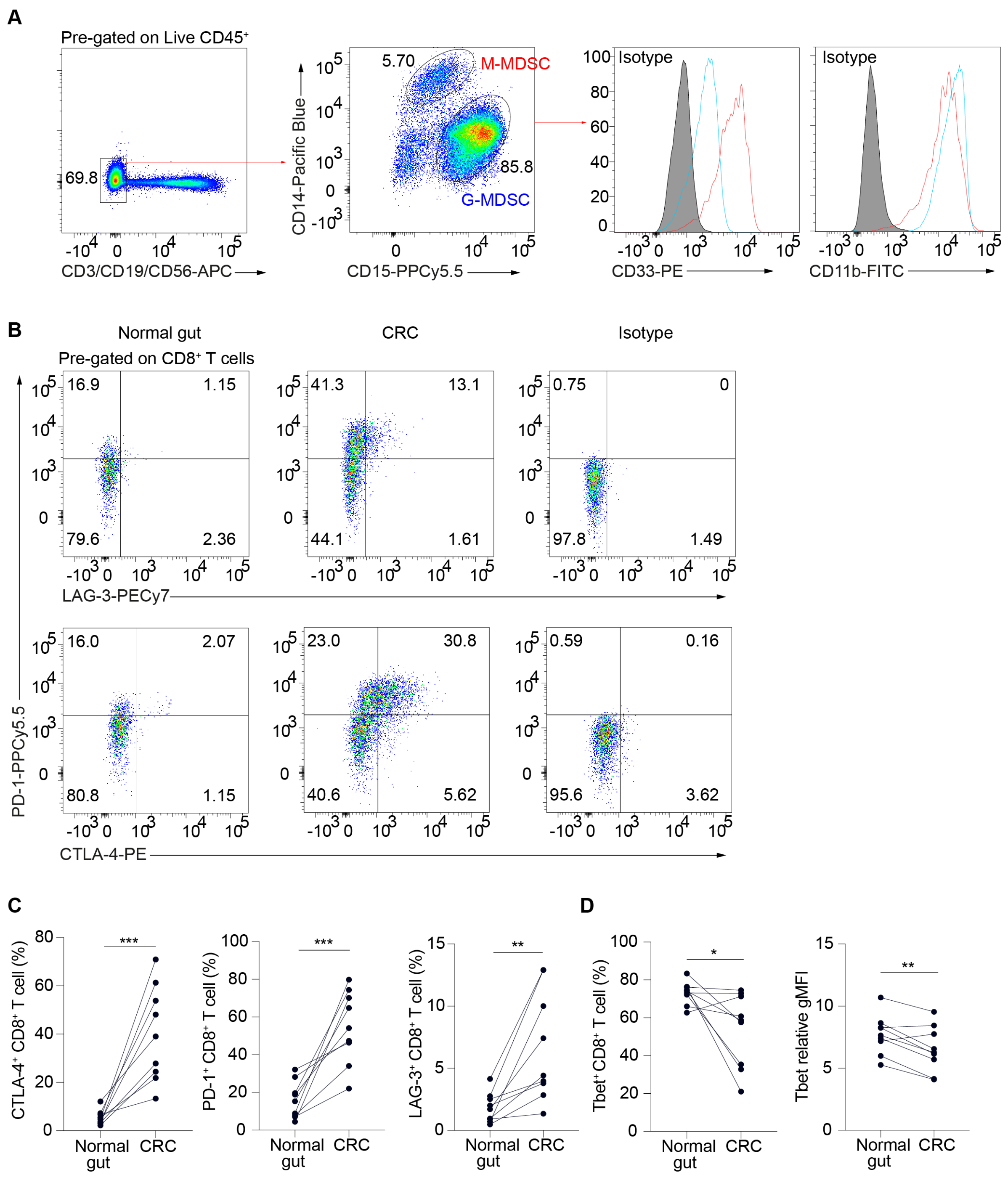

Fig. S12. CD8$^+$ T cells in human CRC show an exhausted phenotype. (**A**) Gating strategy of M-MDSCs and G-MDSCs in human CRC. (**B**) Representative FACS plots showing expression of PD-1, CTLA-4 and LAG-3 on CD8$^+$ T cells from human CRC and adjacent normal gut. (**C** and **D**) Quantification of CTLA-4, PD-1, LAG-3 (C), and Tbet (D) expression on CD8$^+$ T cells in human CRC and adjacent normal gut (n = 9). Relative gMFI, geometric mean fluorescent intensity relative to isotype control. Each pair of dots represents an individual human CRC patient. Data pooled from two

or more independent experiments. Statistical significance determined by paired two-tailed *t*-test. *$P <$ 0.05, **$P < 0.01$, ***$P < 0.001$.

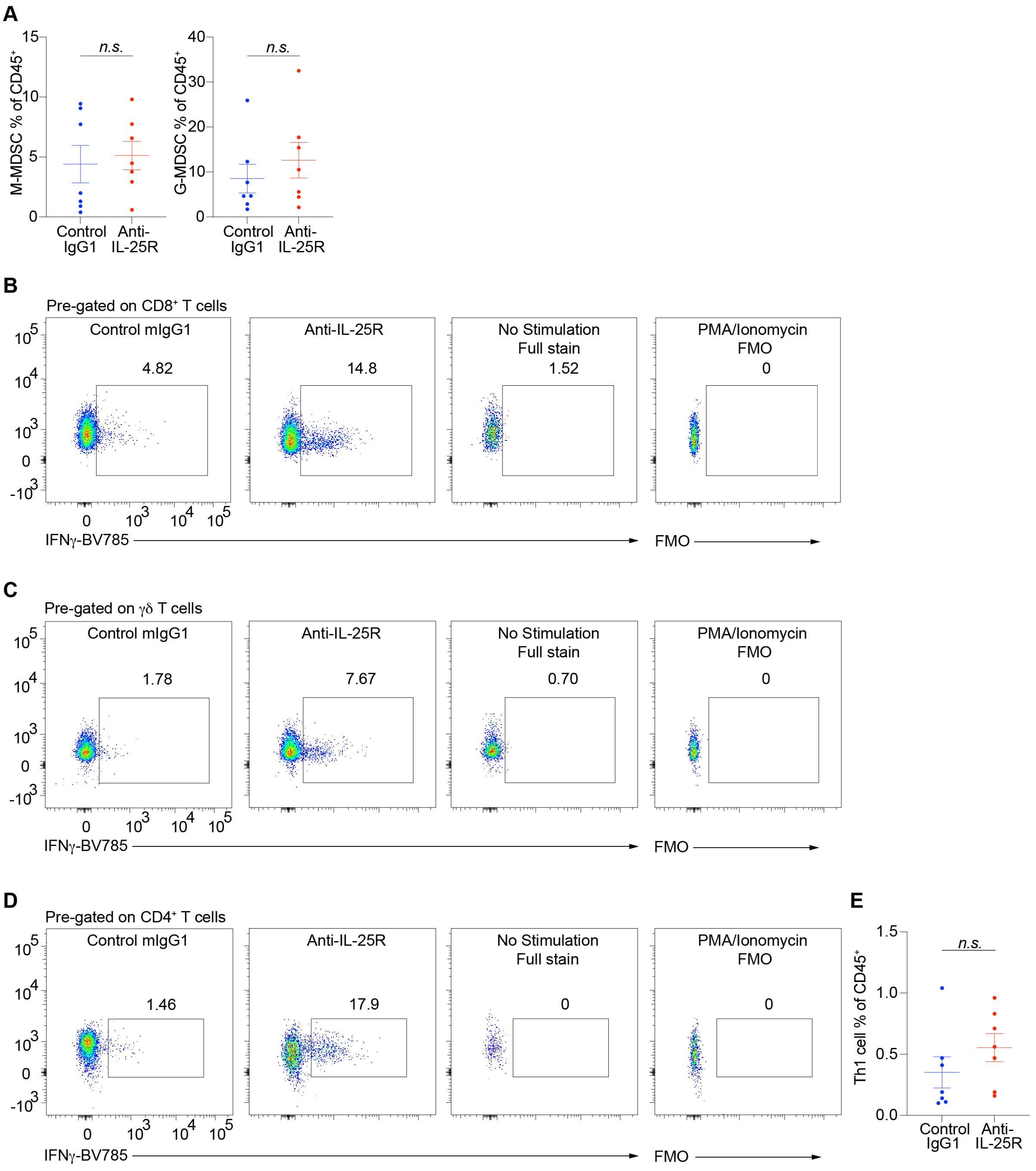

Fig. S13. Therapeutic anti-IL-25R treatment enhances anti-tumor immunity. (**A**) Frequency of M-MDSCs and G-MDSCs in anti-IL-25R or control IgG1-treated *Apc*$^{1322T/+}$ mice (control IgG1, *n* = 7; anti-IL-25R, *n* = 7). (**B** to **D**) Gating strategy of IFNγ expression in tumor CD8^{+} T cells (B), γδ T cells (C), and CD4^{+} T cells (D), in anti-IL-25R or control IgG1-treated *Apc*$^{1322T/+}$ mice. (**E**) Frequency of tumor Th1 cells in anti-IL-25R or control IgG1-treated *Apc*$^{1322T/+}$ mice (control IgG1, *n* = 7; anti-IL-25R, *n* = 7). Data collected from female mice at 11-weeks of age, treated with anti-IL-25R or control IgG1 for 4 weeks (treatment started in adults at 7-weeks of age with established tumors), and pooled

from two independent experiments; error bars show mean ± SEM. Statistical significance determined by unpaired two-tailed *t*-test. *n.s.* non-signifiacnt.

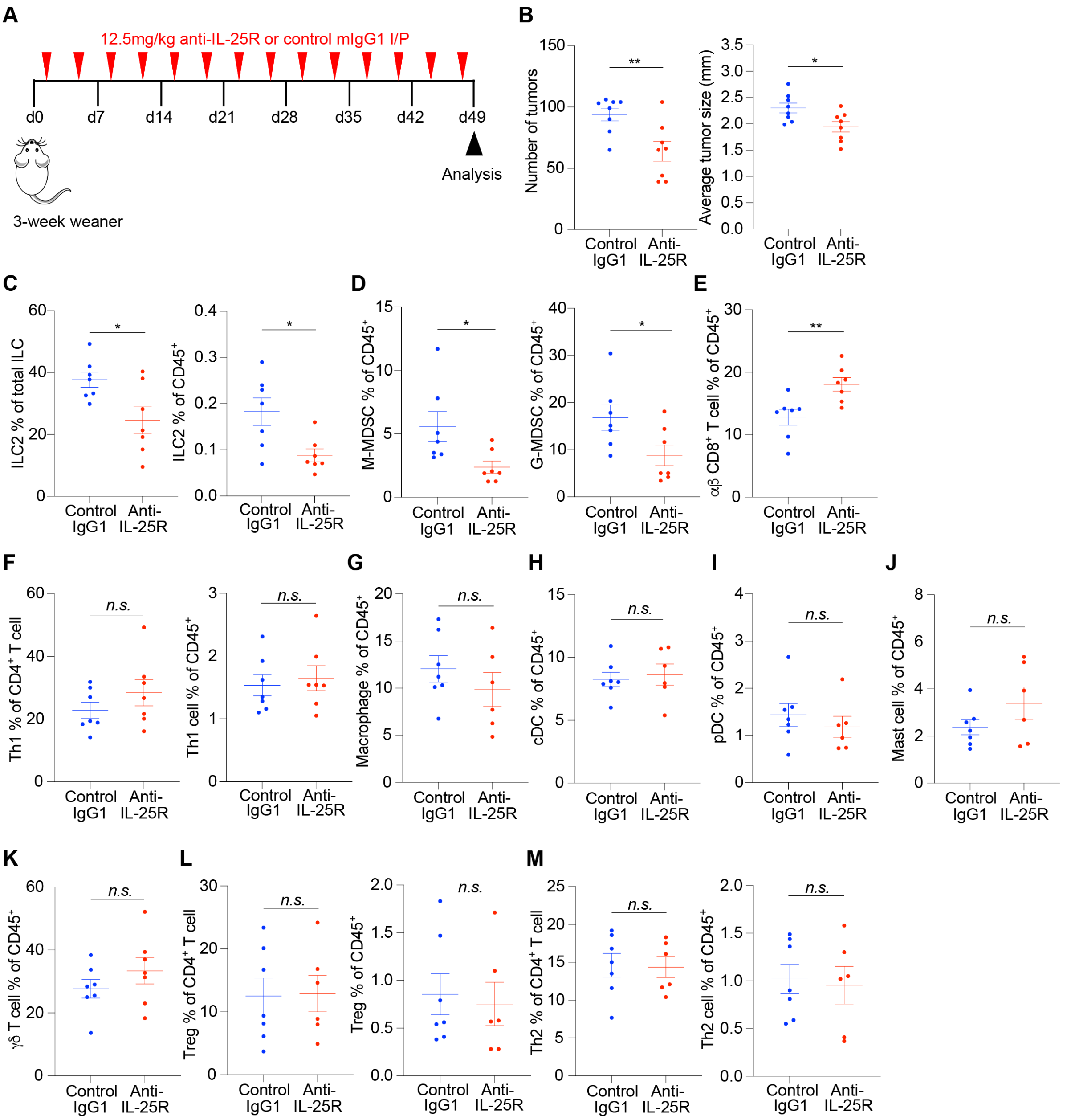

Fig. S14. Prophylactic anti-IL-25R treatment decreases tumor burden. (**A**) Schematic of anti-IL-25R or vehicle treatment starting in young weaner (3-weeks of age) *Apc*$^{1322T/+}$ mice; mice were injected twice per week for seven weeks. (**B**) Number of tumors and average tumor size in anti-IL-25R or control IgG1-treated *Apc*$^{1322T/+}$ mice (control IgG1, n = 8; anti-IL-25R, n = 8). (**C**) Frequency of tumor ILC2s in anti-IL-25R or control IgG1-treated *Apc*$^{1322T/+}$ mice (control IgG1, n = 7; anti-IL-25R, n = 7). (**D** to **F**) Frequency of tumor M-MDSCs and G-MDSCs (D), CD8^{+} T cells (E), and Th1 cells (F), in anti-IL-25R or control IgG1-treated *Apc*$^{1322T/+}$ mice (control IgG1, n = 7; anti-IL-25R, n = 7). (**G** to **M**) Frequency of macrophages (control IgG1, n = 7; anti-IL-25R, n = 6) (G), conventional

dendritic cells (cDC) (control IgG1, $n = 7$; anti-IL-25R, $n = 6$) (H), plasmacytoid dendritic cells (pDC) (control IgG1, $n = 7$; anti-IL-25R, $n = 6$) (I), mast cells (control IgG1, $n = 7$; anti-IL-25R, $n = 6$) (J), γδ T cells (control IgG1, $n = 7$; anti-IL-25R, $n = 7$) (K), Tregs (control IgG1, $n = 7$; anti-IL-25R, $n = 6$) (L), and Th2 cells (control IgG1, $n = 7$; anti-IL-25R, $n = 6$) (M), in anti-IL-25R or control IgG1-treated $Apc^{1322T/+}$ mice. Data collected from female mice at 10-weeks of age, treated with anti-IL-25R or control IgG1 for 7 weeks (treatment started from 3-weeks of age in young weaners), and pooled from two or more independent experiments; error bars show mean ± SEM. Statistical significance determined by unpaired two-tailed *t*-test. *n.s.* non-signifiacnt, $*P < 0.05$, $**P < 0.01$.

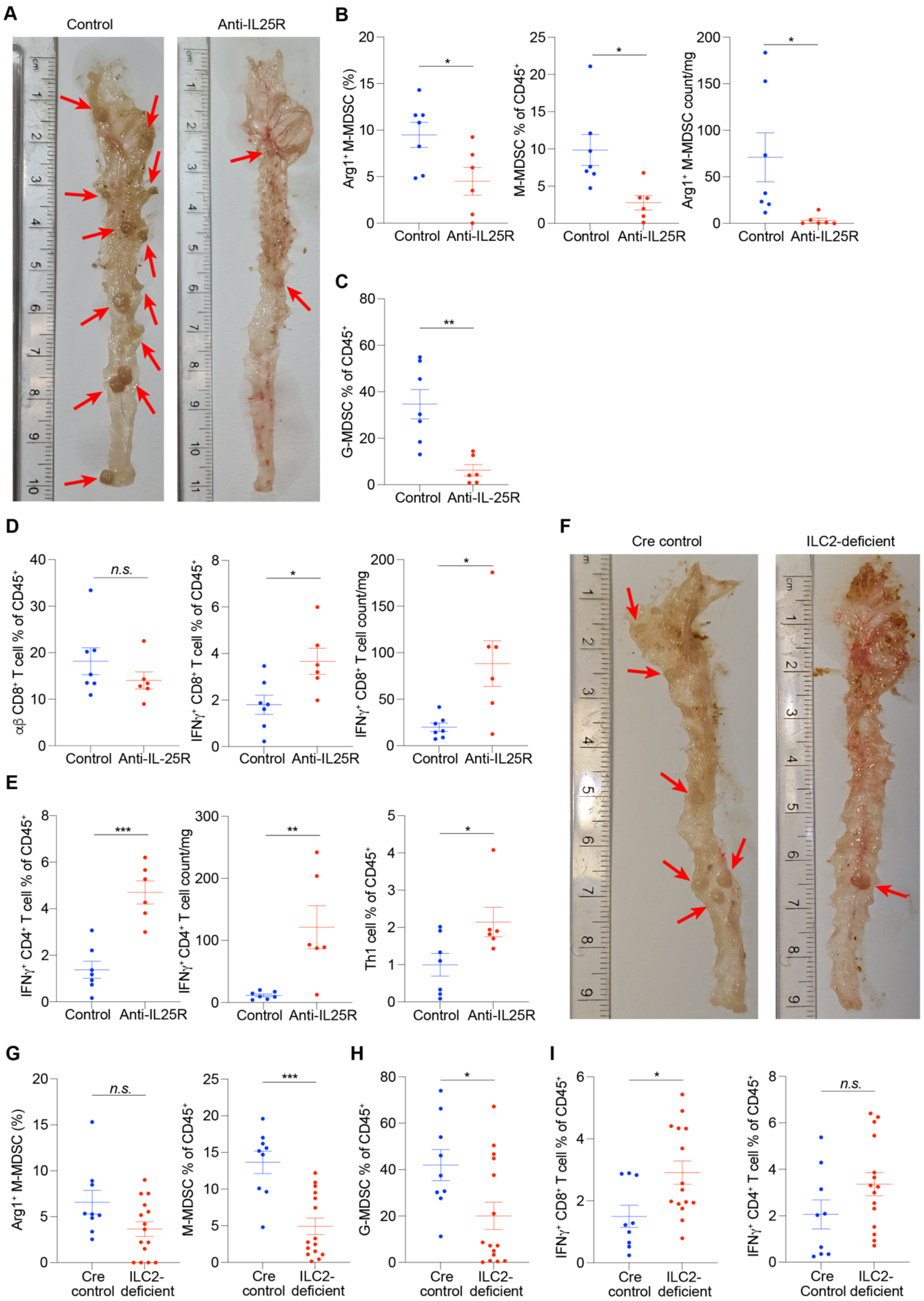

Fig. S15. Blocking the IL-25-ILC2 axis decreases colonic tumor MDSCs. (**A**) Representative fresh whole colon images from anti-IL-25R or control IgG1-treated *Apc*$^{1322T/+}$-DSS mice, red arrow points

to tumors. *Apc*$^{1322T/+}$-DSS mice, dextran sulfate sodium treated *Apc*$^{1322T/+}$ mice. (**B** to **D**) Frequency of colonic tumor M-MDSCs and Arg1 expression (B), and frequency of G-MDSCs (C) and CD8^{+} T cells (D), in anti-IL-25R or control IgG1-treated *Apc*$^{1322T/+}$-DSS mice (control IgG1, $n = 7$; anti-IL-25R, $n = 6$). (**E**) Frequency of tumor Th1 cells and IFNγ^{+} CD4^{+} T cells in anti-IL-25R or control IgG1-treated *Apc*$^{1322T/+}$-DSS mice (control IgG1, $n = 7$; anti-IL-25R, $n = 6$). (**F**) Representative fresh whole colon images from ILC2-deficient (*Rora*$^{f/f}$*Il7r*$^{Cre/+}$) and Cre control (*Rora*$^{+/+}$*Il7r*$^{Cre/+}$) *Apc*$^{1322T/+}$-DSS mice, red arrow points to tumors. (**G** and **H**) Frequency of colonic tumor M-MDSCs and Arg1 expression (G), and frequency of G-MDSCs (H) (Cre control, $n = 9$; ILC2-deficient, $n = 15$), from ILC2-deficient (*Rora*$^{f/f}$*Il7r*$^{Cre/+}$) and Cre control (*Rora*$^{+/+}$*Il7r*$^{Cre/+}$) *Apc*$^{1322T/+}$-DSS mice. (**I**) Frequency of colonic tumor IFNγ^{+} CD8^{+} and CD4^{+} T cells (Cre control, $n = 9$; ILC2-deficient, $n = 15$) from ILC2-deficient and Cre control *Apc*$^{1322T/+}$-DSS mice. Data collected from age-matched female mice treated with DSS and pooled from two or more independent experiments; error bars show mean ± SEM. Statistical significance determined by unpaired two-tailed *t*-test. *n.s.* non-signifiacnt, $*P < 0.05$, $**P < 0.01$, $***P < 0.001$.

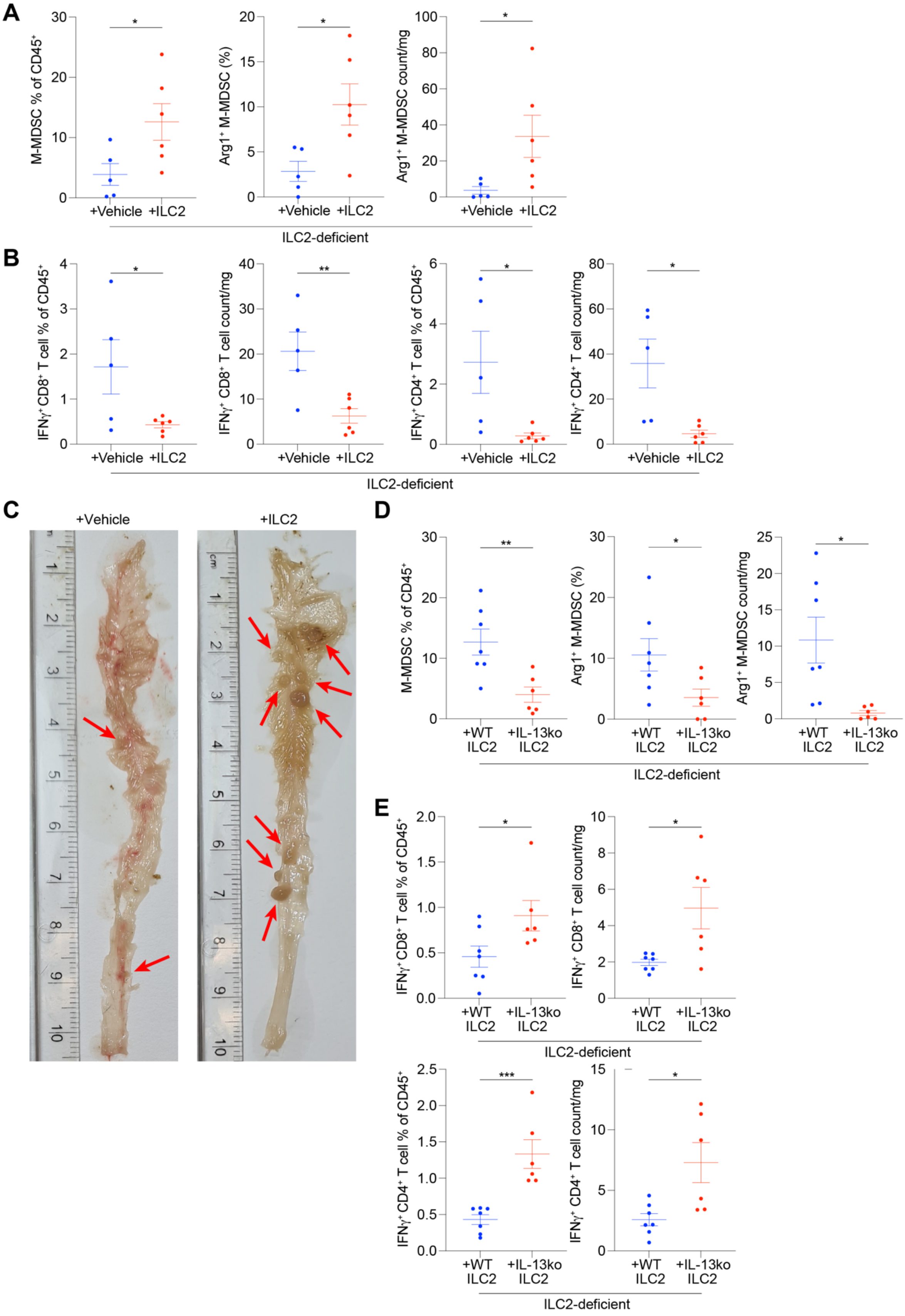

Fig. S16. Adoptive transfer of ILC2s promotes M-MDSCs and suppresses T cell IFNγ expression

(**A** and **B**) Frequency of colonic tumor M-MDSCs and Arg1 expression (A), and frequency of IFNγ^+ $CD8^+$ and $CD4^+$ T cells (B), in ILC2-deficient *Apc*$^{1322T/+}$-DSS mice adoptive transferred with ILC2s or control (Control, $n = 5$; ILC2, $n = 6$). *Apc*$^{1322T/+}$-DSS mice, dextran sulfate sodium treated *Apc*$^{1322T/+}$ mice. (**C**) Representative fresh whole colon images from ILC2-deficient *Apc*$^{1322T/+}$-DSS mice adoptive transferred with ILC2s or control, red arrow points to tumors. (**D** and **E**) Frequency of colonic tumor M-MDSCs and Arg1 expression (D), and frequency of IFNγ^+ $CD8^+$ (top) and $CD4^+$ (bottom) T cells (E), in ILC2-deficient *Apc*$^{1322T/+}$-DSS mice adoptive transferred with wild type or IL-13-deficient (IL-13ko) ILC2s (wild type, $n = 7$; IL-13-deficient, $n = 6$). Data collected from age-matched female mice treated with DSS and pooled from two or more independent experiments; error bars show mean ± SEM. Statistical significance determined by unpaired two-tailed *t*-test. *$P < 0.05$, **$P < 0.01$, ***$P < 0.001$.

Strain	Acquired from	Original publication
Apc^{1322T}	MRC LMB (Mariann Bienz lab)	(*22*)
Il25^{tom}	MRC LMB (produced in house)	Unpublished
C57BL/6Jola	Jackson Labs (Bred in ARES)	RRID: IMSR_JAX:000664
Il7ra^{Cre}	Prof. Hans-Reimer Rodewald	(*69*)
Rora^{flox}	MRC LMB (produced in house)	(*4*)
Il13^{tom}	MRC LMB (produced in house)	(*38*)
CD4^{Cre}	Taconic	(*70*)

Table S1. Mouse strains used in this study

Primer	Sequence
ASEQ8252	TACTCTGGGCTTGGGGTTGCTAAGG
ASEQ8253	GAACTCTTTGATGACCTCCTCGCCC
ASEQ8255	GAATTCCTGCAGCCCAATTCCGATCATATTCAATAACCC
ASEQ8256	AAAGGAGACCACCAGGAAGATGACG
ASEQ8257	TGTCATGGTTTTAGTGTCAG
ASEQ8258	TCAGGAGTATTTTCTGAGGG
ASEQ8259	CATCCCAGGAAGAGGTCAGG
ASEQ8261	CCCCTTTTGTTGGCTAAACC
ASEQ8265	TGTCACATACACATTGTAACTC
ASEQ8266	AAAGAAGCCGTTAGTGTCCC
ASEQ8268	TACAGACAGGCTCCCACATGGACCC
ASEQ8269	CCCCTTTGCCTTTTGTTGTG
ASEQ8270	AGACCCAGCCAAATAAGAGG
ASEQ8271	GCAGGGCTCCAGGGCTTCGG
ASEQ8273	GAGATGCCCTTGATGGTCCC
ASEQ8275	AGACATATGAGTTATGGTGG
ASEQ8287	GTTTGGGAGCTACACAGGCC
ASEQ8291	ACAAGCCACCTGAGACCACG
ASEQ8292	ACTGGCACTGGATGTGTTTC

Table S2. Amplification and sequencing primers used in the generation and validation of *Il25*-tomato gene-targeted mice.
